# GeneSeqToFamily: a Galaxy workflow to find gene families based on the Ensembl Compara GeneTrees pipeline

**DOI:** 10.1093/gigascience/giy005

**Published:** 2018-02-07

**Authors:** Anil S Thanki, Nicola Soranzo, Wilfried Haerty, Robert P Davey

**Affiliations:** Earlham Institute, Norwich Research Park, Norwich NR4 7UZ, UK

**Keywords:** Galaxy, Pipeline, Workflow, Genomics, Comparative Genomics, Homology, Orthology, Paralogy, Phylogeny, Gene Family, Alignment, Compara, Ensembl

## Abstract

**Background:**

Gene duplication is a major factor contributing to evolutionary novelty, and the contraction or expansion of gene families has often been associated with morphological, physiological, and environmental adaptations. The study of homologous genes helps us to understand the evolution of gene families. It plays a vital role in finding ancestral gene duplication events as well as identifying genes that have diverged from a common ancestor under positive selection. There are various tools available, such as MSOAR, OrthoMCL, and HomoloGene, to identify gene families and visualize syntenic information between species, providing an overview of syntenic regions evolution at the family level. Unfortunately, none of them provide information about structural changes within genes, such as the conservation of ancestral exon boundaries among multiple genomes. The Ensembl GeneTrees computational pipeline generates gene trees based on coding sequences, provides details about exon conservation, and is used in the Ensembl Compara project to discover gene families.

**Findings:**

A certain amount of expertise is required to configure and run the Ensembl Compara GeneTrees pipeline via command line. Therefore, we converted this pipeline into a Galaxy workflow, called GeneSeqToFamily, and provided additional functionality. This workflow uses existing tools from the Galaxy ToolShed, as well as providing additional wrappers and tools that are required to run the workflow.

**Conclusions:**

GeneSeqToFamily represents the Ensembl GeneTrees pipeline as a set of interconnected Galaxy tools, so they can be run interactively within the Galaxy's user-friendly workflow environment while still providing the flexibility to tailor the analysis by changing configurations and tools if necessary. Additional tools allow users to subsequently visualize the gene families produced by the workflow, using the Aequatus.js interactive tool, which has been developed as part of the Aequatus software project.

## Introduction

The phylogenetic information inferred from the study of homologous genes helps us to understand the evolution of gene families (also referred to as “orthogroups”) that comprise genes sharing common descent [1]. This plays a vital role in finding ancestral gene duplication events as well as in identifying regions under positive selection within species [2]. In order to investigate these low-level comparisons between gene families, the Ensembl Compara GeneTrees gene orthology and paralogy prediction software suite [3] was developed as a pipeline. The Ensembl GeneTrees pipeline uses TreeBest [4, 5] (part of TreeFam [6]), which implements multiple independent phylogenetic methods and can merge the results into a consensus tree while trying to minimize duplications and deletions relative to a known species tree. This allows TreeBeST to take advantage of the fact that DNA-based methods are often more accurate for closely related parts of trees, while protein-based trees are better at longer evolutionary distances.

The Ensembl GeneTrees pipeline comprises 7 steps, starting from a set of protein sequences and performing similarity searching and multiple large-scale alignments to infer homology among them, using various tools: BLAST [7], hcluster_sg [8], T-Coffee [9], and phylogenetic tree construction tools, including TreeBeST. While these tools are freely available, most are specific to certain computing environments, are only usable via the command line, and require many dependencies to be fulfilled. Therefore, users are not always sufficiently expert in system administration to install, run, and debug the various tools at each stage in a chain of processes. To help ease the complexity of running the GeneTrees pipeline, we employed the Galaxy bioinformatics analysis platform to relieve the burden of managing these system-level challenges.

Galaxy is an open-source framework for running a broad collection of bioinformatics tools via a user-friendly web interface [10]. No client software is required other than a recent web browser, and users are able to run tools singly or aggregated into interconnected pipelines, called “workflows”. Galaxy enables users to not only create but also share workflows with the community. In this way, it helps users who have little or no bioinformatics expertise to run potentially complex pipelines in order to analyze their own data and interrogate results within a single online platform. Furthermore, pipelines can be published in a scientific paper or in a repository such as myExperiment [11] to encourage transparency and reproducibility.

In addition to analytical tools, Galaxy also contains plugins [12] for data visualization. Galaxy visualization plugins may be interactive and can be configured to visualize various data types, for example, bar plots, scatter plots, and phylogenetic trees. It is also possible to develop custom visualization plugins and easily integrate them into Galaxy. As the output of the GeneSeqToFamily workflow is not conducive to human readability, we also provide a data-to-visualization plugin based on the Aequatus software [13]. Aequatus.js [14] is a new JavaScript library for the visualization of homologous genes that we extracted from the standalone Aequatus software. It provides a detailed view of gene structure across gene families, including shared exon information within gene families alongside gene tree representations. It also shows details about the type of interrelation event that gave rise to the family, such as speciation, duplication, and gene splits.

## Methods

The GeneSeqToFamily workflow has been developed to run the Ensembl Compara software suite within the Galaxy environment (Galaxy, RRID:SCR_006281), combining various tools alongside preconfigured parameters obtained from the Ensembl Compara pipeline to produce gene trees. Among the tools used in GeneSeqToFamily (listed in Table 1), some were existing tools in the Galaxy ToolShed [15], such as NCBI BLAST (NCBI BLAST, RRID:SCR_004870), TranSeq (Transeq, RRID:SCR_015647), Tranalign, and various format converters. Additional tools that are part of the pipeline were developed at the Earlham Institute (EI) and submitted to the ToolShed, that is, *BLAST parser, hcluster_sg, hcluster_sg parser, T-Coffee, TreeBeST best*, and *Gene Alignment and Family Aggregator.* Finally, we developed helper tools that are not part of the workflow itself but aid the generation of input data for the workflow, and these are also in the ToolShed, i.e. *Get features by Ensembl ID, Get sequences by Ensembl ID, Select longest CDS per gene, ETE species tree generator*, and *GeneSeqToFamily preparation*.

**Table 1:** Galaxy tools used in the workflow

			Developed at Earlham Institute		Toolsheds
Tool name	Tool ID	Version	Tool	Wrapper	reference
Get sequences by Ensembl ID	get_sequences	0.1.2	Yes	Yes	[17]
Get features by Ensembl ID	get_feature_info	0.1.2	Yes	Yes	[18]
Select longest coding sequence per gene	ensembl_longest_cds_per_gene	0.0.2	Yes	Yes	[19]
ETE species tree generator	ete_species_tree_generator	3.0.0b35	Yes	Yes	[20]
GeneSeqToFamily preparation	gstf_preparation	0.4.0	Yes	Yes	[21]
Transeq	EMBOSS: transeq101	5.0.0	No	No	[22]
NCBI BLAST+ makeblastdb	ncbi_makeblastdb	0.2.01	No	No	[23]
NCBI BLAST+ blastp	ncbi_blastp_wrapper	0.2.01	No	No	[23]
BLAST parser	blast_parser	0.1.2	Yes	Yes	[24]
hcluster_sg	hcluster_sg	0.5.1.1	No	Yes	[25]
hcluster_sg parser	hcluster_sg_parser	0.2.0	Yes	Yes	[26]
Filter by FASTA IDs	filter_by_fasta_ids	1.0	No	No	[27]
T-Coffee	t_coffee	11.0.8	No	Yes	[28]
Tranalign	EMBOSS: tranalign100	5.0.0	No	No	[22]
TreeBeST best	treebest_best	1.9.2	No	Yes	[29]
Gene Alignment and Family Aggregator	gafa	0.3.0	Yes	Yes	[30]
Unique	tp_sorted_uniq	1.1.0	No	No	[31]
FASTA-to-Tabular	fasta2tab	1.1.0	No	No	[32]
UniProt ID mapping and retrieval	uniprot_rest_interface	0.1	No	No	[33]

**Figure 1:**
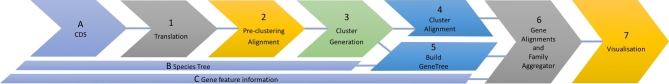
Overview of the GeneSeqToFamily workflow.

**Figure 2:**
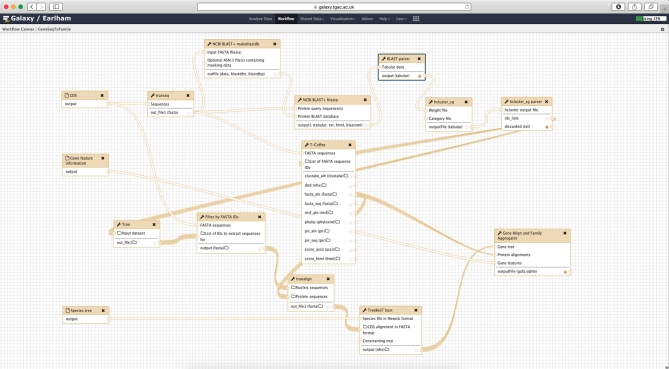
Screenshot from the Galaxy Workflow Editor showing the GeneSeqToFamily workflow.

The workflow comprises 7 main steps (see Figure 1), starting with translation from input coding sequences (CDS) to protein sequences, finding subsequent pairwise alignments of those protein sequences using BLASTP, and then the generation of clusters from the alignments using hcluster_sg. The workflow then splits into 2 simultaneous paths, whereby in one path it performs the multiple sequence alignment (MSA) for each cluster using T-Coffee (T-Coffee, RRID:SCR_011818), while in the other it generates a gene tree with TreeBeST taking the cluster alignment and a species tree as input. Finally, these paths merge to aggregate the MSA, the gene tree, and the gene feature information (eg, transcripts, exons) into an SQLite [16] database for visualization and downstream reuse. All the workflow and data preparation steps are shown in Figure 2 and explained in detail below.

## Data generation and preparation

We developed a number of tools that assist in preparing the datasets needed by the workflows.

### 

#### Ensembl REST API tools

Galaxy tools were developed that use the Ensembl REST API [34] to retrieve sequence information (*Get sequences by Ensembl ID*) and feature information (*Get features by Ensembl ID*) by Ensembl ID from the Ensembl service. REST (REpresentational State Transfer) is an architecture style for designing networked applications [35] that encourages the use of standardized HTTP technology to send and receive data between computers. As such, these tools are designed to help users to retrieve existing data from Ensembl rather than requiring them to manually download datasets to their own computers and then subsequently uploading them into the workflow.

#### ETE tools

We have developed the *ETE species tree generator* Galaxy tool, which uses the ETE toolkit [36] to generate a species tree from a list of species names or taxon IDs through the NCBI Taxonomy.

## GeneSeqToFamily workflow

### 0. GeneSeqToFamily preparation

Before GeneSeqToFamily can be run, a data preparation step must be carried out. We developed a tool called *GeneSeqToFamily preparation* to preprocess the input datasets (gene feature information and CDS) for the GeneSeqToFamily workflow. It converts a set of gene feature information files in GFF3 [37] and/or JavaScript Object Notation (JSON) [38] format to an SQLite database. It also modifies all CDS FASTA header lines by appending the species name to the transcript identifier, as required by *TreeBeST best*. It can also retain only the longest CDS sequence for each gene, as done in the GeneTrees pipeline.

We decided to use an SQLite database to store the gene feature information because the GFF3 format has a relatively inconvenient and unstructured additional information field (9th column) and because searching is much faster and more memory efficient in a database than in a text file like JSON or GFF3, especially when dealing with feature information for multiple large genomes.

### 1. CDS translation

#### Transeq

Transeq, part of the European Molecular Biology Open Software Suite (EMBOSS) (EMBOSS, RRID:SCR_008493) [39], is a tool to generate 6-frame translation of nucleic acid sequences to their corresponding peptide sequences. Here, we use Transeq to convert a CDS to protein sequences in order to run BLASTP (BLASTP, RRID:SCR_001010) and find protein clusters. However, since downstream tools in the pipeline, such as TreeBeST, require nucleotide sequences to generate a gene tree, the protein sequences cannot be directly used as workflow input and are instead generated with Transeq.

### 2. Preclustering alignment

#### BLAST

This workflow uses the BLAST wrappers [40] developed to run BLAST+ tools within Galaxy. BLASTP is run over the set of sequences against the database of the same input, as is the case with BLAST-all, in order to form clusters of related sequences.

#### BLAST parser

BLAST parser is a small Galaxy tool to convert the BLAST output into the input format required by hcluster_sg. It takes the BLAST 12-column output [41] as input and generates a 3-column tabular file, comprising the BLAST query, the hit result, and the edge weight. The weight value is simply calculated as minus log_10_ of the BLAST e-value divided by 2, replacing this with 100 if this value is greater than 100. It also removes the self-matching BLAST results and lets the user filter out non-Reciprocal Best Hits.

### 3. Cluster generation

#### hcluster_sg

hcluster_sg performs clustering for sparse graphs. It reads an input file that describes the similarity between 2 sequences, and iterates through the process of grouping 2 nearest nodes at each iteration. hcluster_sg outputs a single list of gene clusters, each comprising a set of sequence IDs present in that cluster. This list needs to be reformatted using the *hcluster_sg parser* tool in order to be suitable for input into T-Coffee and TreeBeST (see below).

#### hcluster_sg parser

hcluster_sg parser converts the hcluster_sg output into a collection of lists of IDs, 1 list for each cluster. Each of these clusters will then be used to generate a gene tree via TreeBeST. The tool can also filter out clusters with a number of elements outside a specified range. The IDs contained in all discarded clusters are collected in separate output dataset. Since TreeBeST requires at least 3 genes to generate a gene tree, we configured the tool to filter out clusters with less than 3 genes.

*Filter by FASTA IDs*, which is available from the Galaxy ToolShed, is used to create separate FASTA files using the sequence IDs listed in each gene cluster.

### 4. Cluster alignment

#### T-Coffee

T-Coffee is a MSA package but can also be used to combine the output of other alignment methods (Clustal, MAFFT, Probcons, MUSCLE) into a single alignment. T-Coffee can align both nucleotide and protein sequences [9]. We use it to align the protein sequences in each cluster generated by hcluster_sg.

We modified the Galaxy wrapper for T-Coffee to take a single FASTA (as normal) and an optional list of FASTA IDs to filter. If a list of IDs is provided, the wrapper will pass only those sequences to T-Coffee, which will perform the MSA for that set of sequences, thus removing the need to create thousands of intermediate Galaxy datasets.

### 5. Gene tree construction

#### Tranalign

Tranalign [39] is a tool that reads a set of nucleotide sequences and a corresponding aligned set of protein sequences and returns a set of aligned nucleotide sequences. Here, we use it to generate CDS alignments of gene sequences using the protein alignments produced by T-Coffee.

#### TreeBeST “best”

TreeBeST (Tree Building guided by Species Tree) is a tool to generate, manipulate, and display phylogenetic trees and can be used to build gene trees based on a known species tree.

The “best” command of TreeBeST builds 5 different gene trees from a FASTA alignment file using different phylogenetic algorithms, then merges them into a single consensus tree using a species tree as a reference. In GeneSeqToFamily, *TreeBeST “best”* uses the nucleotide MSAs generated by Tranalign (at least 3 sequences are required) and a user-supplied species tree in Newick format [42] (either produced by a third-party software or through the *ETE species tree generator* data preparation tool, described above) to produce a GeneTree for each family, represented also in Newick format. The resulting GeneTree also includes useful annotations specifying phylogenetic information of events responsible for the presence/absence of genes, for example, “S” means speciation event, “D” means duplication, and “DCS” denotes the duplication score.

### 6. Gene alignment and family aggregation

#### Gene alignment and family aggregator 

Gene alignment and family aggregator (*GAFA*) is a Galaxy tool that generates a single SQLite database containing the gene trees and MSAs, along with gene features, in order to provide a reusable, persistent data store for visualization of synteny information with Aequatus. *GAFA* requires gene trees in Newick format, the protein MSAs in fasta_aln format from *T-Coffee*, and gene feature information generated with the *GeneSeqToFamily preparation* tool.

**Figure 3:**
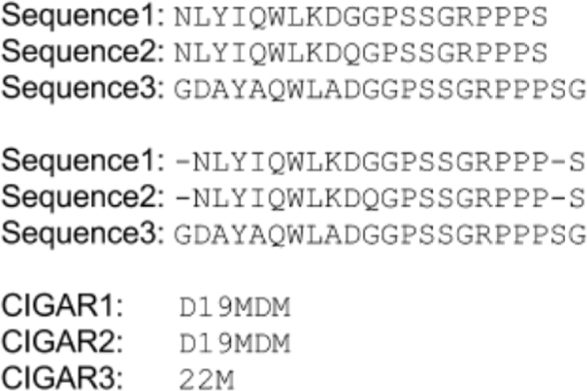
Showing how CIGAR for multiple sequence alignment is generated.

Internally, *GAFA* converts each MSA from fasta_aln format to a simple CIGAR string [43]. An example of CIGAR strings for aligned sequences is shown in Figure 3, in which each CIGAR string changes according to other sequences.

**Figure 4:**
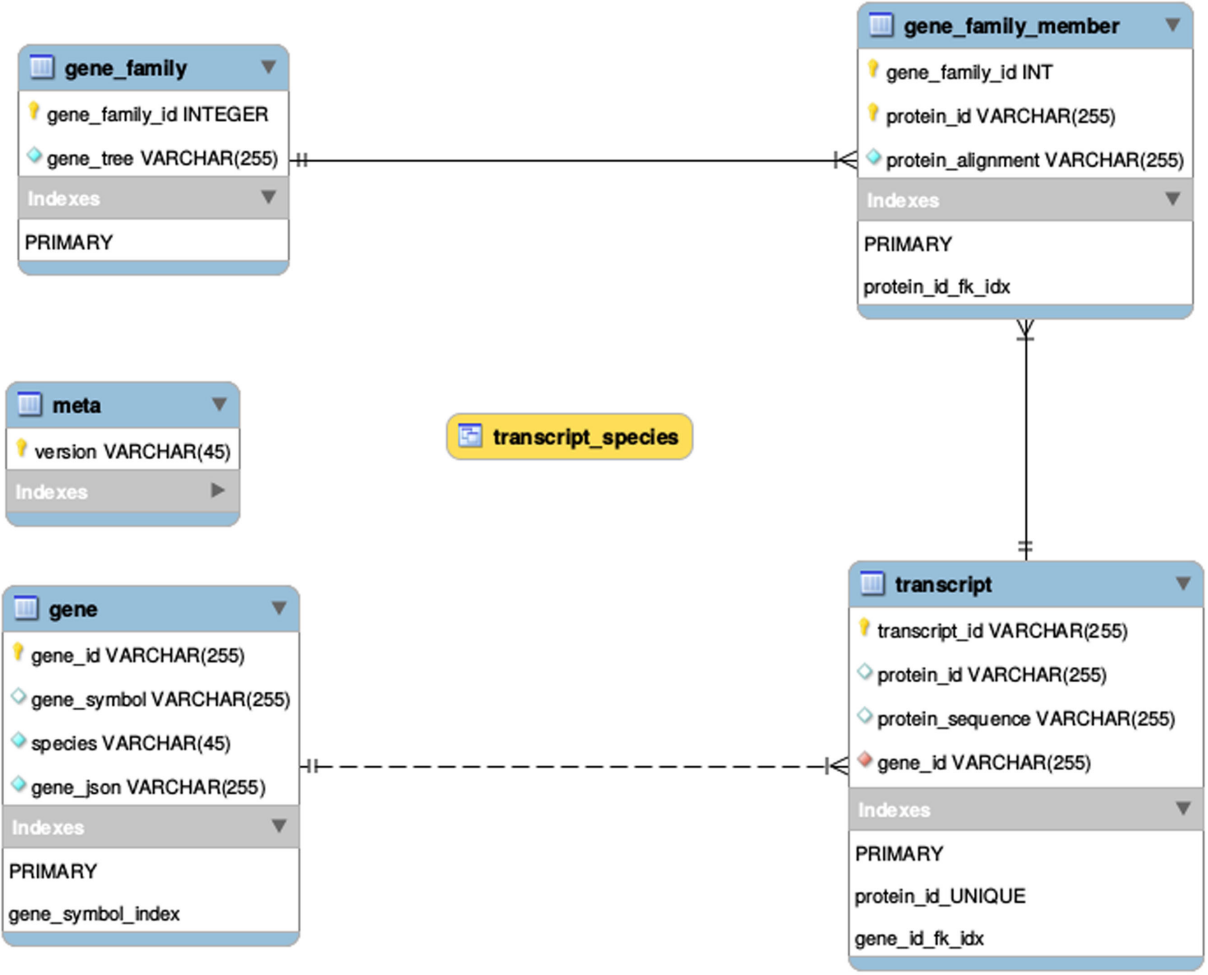
Schema of the gene alignment and family aggregator (GAFA) SQLite database, where transcript_species is a database view.

The simple schema [44] for the generated SQLite database is shown in Figure 4.

### 7. Visualization

#### Aequatus visualization plugin

The SQLite database generated by the GAFA tool can be rendered using a new visualization plugin, Aequatus.js. The Aequatus.js library, developed as part of the Aequatus project, has been configured to be used within Galaxy to visualize homologous gene structure and gene family relationships. This allows users to interrogate not only the evolutionary history of the gene family but also the structural variation (exon gain/loss) within genes across the phylogeny. Aequatus.js is available to download from GitHub [44], as visualization plugins cannot yet be submitted to the Galaxy ToolShed.

## Finding homology information for orphan genes

**Figure 5:**
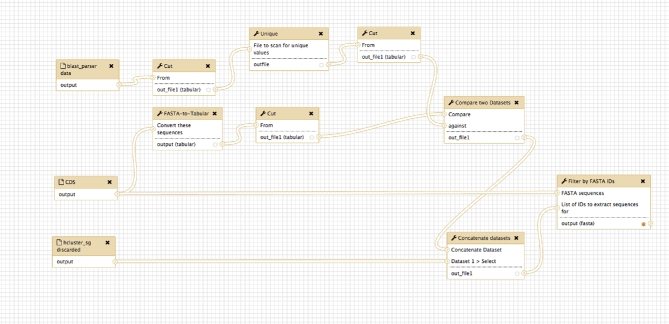
Screenshot from the Galaxy Workflow Editor showing the orphan gene finding workflow.

Although the GeneSeqToFamily workflow will assign most of the genes to orthogroups, many genes within a species might appear to be unique without homologous relationship to any other genes from other species. This observation could be the consequence of the parameters selected, choice of species, or incomplete annotations. This could also reflect real absence of homology, such as for rapidly evolving gene families. In addition to the GeneSeqToFamily workflow, we also developed 2 associated workflows to further annotate these genes by:

1) Retrieving a list of orphan genes from the GeneSeqToFamily workflow (see Figure 5) as follows:
  a) Find the IDs of the sequences present in the input CDS of the GeneSeqToFamily workflow but not in the result of *BLAST parser* from the same workflow.
  b) Add to this list the IDs of the sequences discarded by *hcluster_sg parser*.
  c) From the input CDS dataset, retrieve the respective sequence for each CDS ID (from the step above) using *Filter by FASTA IDs*.

These unique CDS can be fed into the SwissProt workflow below to find homologous genes in other species.

**Figure 6:**
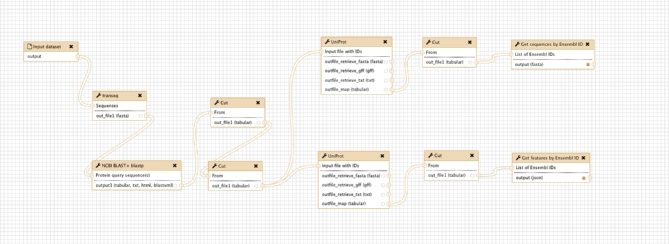
Screenshot from the Galaxy Workflow Editor showing the SwissProt workflow.

2) Finding homologous genes for some genes of interest using SwissProt (see Figure 6) as follows:
  a) Translate CDS into protein sequences using *Transeq*.
  b) Run BLASTP for the protein sequences against the SwissProt database (from NCBI).
  c) Extract UniProt IDs from these BLASTP results, using the preinstalled Galaxy tool *Cut columns from a table* (tool id *Cut1*).
  d) Retrieve Ensembl IDs (representing genes and/or transcripts) for each UniProt ID using *UniProt ID mapping and retrieval*.
  e) Get genomic information for each gene ID and CDS for each transcript ID from the core Ensembl database using *Get features by Ensembl ID* and *Get sequences by Ensembl ID*, respectively.

The results from this second workflow can be subsequently used as input to GeneSeqToFamily for familial analysis.

## Results

To validate the biological relevance of results from the GeneSeqToFamily workflow, we analyzed a small set of 23 homologous genes (1 transcript per gene) from *Pan troglodytes* (chimpanzee), *Homo sapiens* (human), *Rattus norvegicus* (rat), *Mus musculus* (mouse), *Sus scrofa* (pig), and *Canis familiaris* (domesticated dog). These genes are a combination of those found in 3 gene families, that is, monoamine oxidases (MAO A and B), insulin receptor (INSR), and BRCA2, and were chosen because they are present in all 6 species yet distinct from each other.

Before running the workflow, feature information and CDS for the selected genes were retrieved from the core Ensembl database using the helper tools described above (*Get features by Ensembl ID* and *Get sequences by Ensembl ID*, respectively), and CDS were filtered to keep the longest CDS per gene. A species tree was generated using *ETE species tree generator*, and inputs were prepared with *GeneSeqToFamily preparation*.

**Figure 7:**
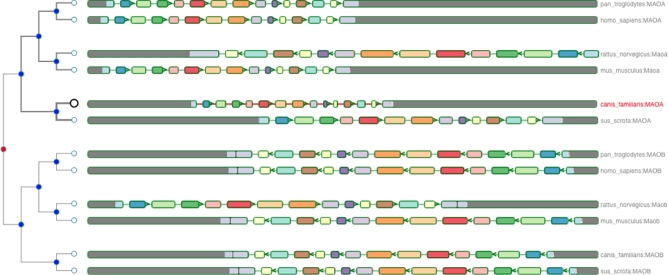
Homologous genes of monoamine oxidase (MAO) of *Canis familiaris* from *Mus musculus, Pan troglodytes, Homo sapiens, Rattus norvegicus, Sus scrofa*, and *Canis familiaris*.

**Figure 8:**
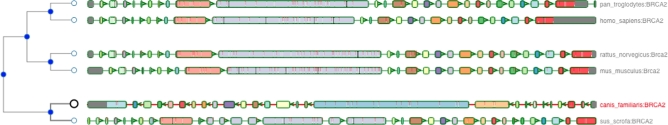
Homologous genes of BRCA2 of *Canis familiaris* from *Mus musculus, Pan troglodytes, Homo sapiens, Rattus norvegicus*, and *Sus scrofa*.

**Figure 9:**
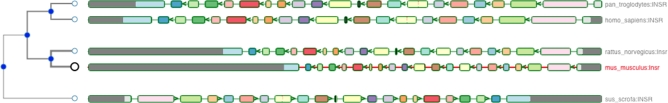
Homologous genes of insulin receptor (INSR) of *Mus musculus* from *Pan troglodytes, Homo sapiens, Rattus norvegicus*, and *Sus scrofa*.

We ran the GeneSeqToFamily workflow on these data using the default parameters of the Ensembl Compara pipeline (Table 2, experiment D). This workflow generated 3 different gene trees, each matching exactly 1 gene family. Figures 7–9 show the resulting gene trees for MAO, BRCA2, and INSR gene families. Different colors of the nodes in each gene tree on the left-hand side of the visualization highlight potential evolutionary events, such as speciation, duplication, and gene splits. Homologous genes showing shared exons use the same color in each representation, including insertions (black blocks) and deletions (red lines). The GeneTrees for these genes are already available in Ensembl; we used them to validate our findings [45–48]. Our gene trees agree perfectly with the Ensembl GeneTrees, showing that the workflow generates biologically valid results. We have provided the underlying data for this example along with the submitted workflow in figshare [49].

**Table 2:** Set of parameters used in BLASTP and hcluster_sg to compare results

		Parameter set					
Tool	Parameter	A	B	C	D	E	F
BLASTP	Expectation value cutoff	1e-03	1e-03	1e-03	1e-10	1e-10	1e-10
	Query coverage per hsp	0	0	90	0	0	90
hcluster_sg	Minimum edge weight	0	20	0	0	20	20
	Minimum edge density between a join	0.34	0.50	0.34	0.34	0.50	0.50

BLASTP was configured with maximum number of HSPs set to 1, and hcluster_sg with single link clusters set to “no” and maximum size set to 500.

We also studied the impact of the most important tool parameters on the gene families reconstructed by the workflow by running it on larger datasets, in particular, the reference proteomes of 754,149 sequences from 66 species established by the Quest for Orthologs (QfO) consortium [50]. We ran GeneSeqToFamily (up to the hcluster_sg step, where gene families are determined) with various sets of parameters (shown in Table 2) and performed statistical analysis on the resulting gene families (Table 3). Our results show that the number of gene families can vary quite distinctly with different BLASTP and hcluster_sg parameters. Stringent parameters (Parameter Set F) result in a large number of smaller families, while relaxed parameters (Parameter Set A) generate a small number of larger families, which may include distantly related genes. The parameters used by Ensembl Compara as default are shown in Parameter Set D.

**Table 3:** Results of the GeneSeqToFamily workflow run with 7 different sets of parameters, the complete list of which are shown in Table 2

Summary						
Analysis	A	B	C	D	E	F
Number of genes	754,149	754,149	754,149	754,149	754,149	754,149
Number of families	58,272	74,252	83,900	63,289	74,309	79,879
Number of larger families (>200)	435	168	56	350	167	46
Number of smaller families (<3)	30,563	40,530	44,295	33,308	40,579	41,794
Families (>3 and <200)	27,274	33,556	39,548	29,628	33,562	38,039
Largest family size	615	567	556	652	561	527
Average family size	11.38	7.36	5.36	10.04	7.35	5.09

We also performed benchmarking using the QfO benchmarking service [50]. QfO benchmarking focuses on assessing the accuracy of a tool to predict 1-to-1 orthology, while the GeneSeqToFamily workflow focuses on whole gene families, regardless of the type of homology among the members of a gene family. GeneSeqToFamily performs comparably to other tools benchmarked in QfO, even surpassing them for True Positive ortholog discovery in some parameter spaces. However, we found issues with the QfO service recording 1-to-many orthologs as false positives, hence reducing our overall specificity. Additional information about the corresponding results of benchmarking is available in Additional File 1.

## Conclusion

The ultimate goal of the GeneSeqToFamily is to provide a user-friendly workflow to analyze and discover homologous genes and their corresponding gene families using the Ensembl Compara GeneTrees pipeline within the Galaxy framework, where users can interrogate genes of interest without using the command-line while still providing the flexibility to tailor analysis by changing configurations and tools if necessary. We have shown it to be an accurate, robust, and reusable method to elucidate and analyze potentially large numbers of gene families in a range of model and nonmodel organisms. The workflow stores the resulting gene families into an SQLite database, which can be visualized using the Aequatus.js interactive tool, as well as shared as a complete reproducible container for potentially large gene family datasets.

We invite the Galaxy community to undertake their own analyses and feedback improvements to various tools, and publish successful combinations of parameters used in the GeneSeqToFamily workflow to achieve better gene families for their datasets. We encourage this process by allowing users to share their own version of GeneSeqToFamily workflow for appraisal by the community.

### Future directions

In terms of core workflow functionality, we would like to incorporate pairwise alignment between pairs of genes for closely related species in addition of the MSA for the gene family, which will help users to compare orthologs and paralogs in greater detail.

We also plan to explicitly include the PantherDB resources [51]. Protein ANalysis THrough Evolutionary Relationships (PANTHER) is a classification system to characterize known proteins and genes according to family, molecular function, biological process, and pathway. The integration of PantherDB with GeneSeqToFamily will enable the automation of gene family validation and add supplementary information about those gene families, which could in turn be used to further validate novel genomics annotation.

Finally, we intend to add the ability to query the *GAFA* SQLite database using keywords in order to make it easy for users to find gene trees that include their genes of interest without needing to delve into the database itself.

## Availability and requirements

**Project name:** GeneSeqToFamily

**Project home page:** https://github.com/TGAC/earlham-galaxytools/tree/master/workflows/GeneSeqToFamily.

**Archived version: 0.1.0**

**Operating system(s):** Platform independent

**Programming language:** JavaScript, Perl, Python, XML, SQL

**Other Requirements:** Web Browser; for development: Galaxy

**Any restrictions to use by non-academics:** None

**License:** The MIT License (https://opensource.org/licenses/MIT)

## Availability of supporting data

The example files and additional datasets supporting the results of this article are available in figshare [49]. A virtual image for Galaxy with necessary tools and installed workflows is available at Earlham repos [52]. Snapshots of the supporting data and code are hosted in the *GigaScience* GigaDB repository [53].

## Additional files

Table S1: Examples of ortholog pairs that are counted as False Positives by QfO benchmarking but are considered orthologs in the Ensembl Compara database.

Table S2: Set of parameters used in BLASTP and hcluster_sg to compare results. BLASTP was configured with maximum number of HSPs set to 1, hcluster_sg with single link clusters set to “no,” and maximum size set to 500.

Figure S1: Showing results for benchmarking on Quest for Orthologs using parameters shown in Parameter Set A http://orthology.benchmarkservice.org/cgi-bin/gateway.pl?f=CheckResults&p1=2569682351ea7dfff3d5b083.

Figure S2: Showing results for benchmarking on Quest for Orthologs using parameters shown in Parameter Set B http://orthology.benchmarkservice.org/cgi-bin/gateway.pl?f=CheckResults&p1=1038fba4ba15c369b3d25541.

Figure S3: Showing results for benchmarking on Quest for Orthologs using parameters shown in Parameter Set C http://orthology.benchmarkservice.org/cgi-bin/gateway.pl?f=CheckResults&p1=ec4d223d24e0a7f54edd3692.

Figure S4: Showing results for benchmarking on Quest for Orthologs using parameters shown in Parameter Set D http://orthology.benchmarkservice.org/cgi-bin/gateway.pl?f=CheckResults&p1=dc81a95f182f5b5bee2dab3f.

Figure S5: Showing results for benchmarking on Quest for Orthologs using parameters shown in Parameter Set E http://orthology.benchmarkservice.org/cgi-bin/gateway.pl?f=CheckResults&p1=9d35f843bcae077e917a6452.

Figure S6: Showing results for benchmarking on Quest for Orthologs using parameters shown in Parameter Set F http://orthology.benchmarkservice.org/cgi-bin/gateway.pl?f=CheckResults&p1=0cbd5a0267b87491252348d6.

## Competing interests

All authors report no competing interests.

## Supplementary Material

GIGA-D-17-00074_Original_Submission.pdf

GIGA-D-17-00074_Revision_1.pdf

GIGA-D-17-00074_Revision_2.pdf

Response_to_Reviewer_Comments_Original_Submission.docx

Response_to_Reviewer_Comments_Revision_1.docx

Reviewer_1_Report_(Original_Submission) -- Kristoffer Forslund 24 Apr 2017 Reviewed

Reviewer_1_Report_(Revision_1) -- Kristoffer Forslund 10 Aug 2017 Reviewed

Reviewer_1_Report_(Revision_2) -- Kristoffer Forslund 03 Jan 2018 Reviewed

Reviewer_2_Report_(Original_Submission) -- Yvan Le Bras 28 Apr 2017 Reviewed

Supplemental material
