# Supplementary material for: GeneSeqToFamily: a Galaxy workflow to find gene families based on the Ensembl Compara GeneTrees pipeline
Source: Gigascience. 2018 Feb 7;7(3):giy005. doi: 10.1093/gigascience/giy005 (PMC5863215; doi:10.1093/gigascience/giy005)
Supplement: GIGA-D-17-00074_Revision_2.pdf [file giy005_giga-d-17-00074_revision_2.pdf]

GigaScience

GeneSeqToFamily: a Galaxy workflow to find gene families based on the Ensembl Compara GeneTrees

--Manuscript Draft--

**Manuscript Number:**	GIGA-D-17-00074R2
**Full Title:**	GeneSeqToFamily: a Galaxy workflow to find gene families based on the Ensembl Compara GeneTrees
**Article Type:**	Technical Note
**Funding Information:**	Biotechnology and Biological Sciences Research Council (BBSRC strategic funds) — Mr. Anil S Thanki, Dr Wilfried Haerty, Dr Robert P Davey<br>Biotechnology and Biological Sciences Research Council (BBSRC Biomathematics and Bioinformatics Training fund (2014).) — Dr Nicola Soranzo
**Abstract:**	Background<br>Gene duplication is a major factor contributing to evolutionary novelty, and the contraction or expansion of gene families has often been associated with morphological, physiological and environmental adaptations. The study of homologous genes helps us to understand the evolution of gene families. It plays a vital role in finding ancestral gene duplication events as well as identifying genes that have diverged from a common ancestor under positive selection. There are various tools available, such as MSOAR, OrthoMCL and HomoloGene, to identify gene families and visualise syntenic information between species, providing an overview of syntenic regions evolution at the family level. Unfortunately, none of them provide information about structural changes within genes, such as the conservation of ancestral exon boundaries amongst multiple genomes. The Ensembl GeneTrees computational pipeline generates gene trees based on coding sequences and provides details about exon conservation, and is used in the Ensembl Compara project to discover gene families.<br><br>Findings<br>A certain amount of expertise is required to configure and run the Ensembl Compara GeneTrees pipeline via command line. Therefore, we have converted the command line Ensembl Compara GeneTrees pipeline into a Galaxy workflow, called GeneSeqToFamily, and provided additional functionality. This workflow uses existing tools from the Galaxy ToolShed, as well as providing additional wrappers and tools that are required to run the workflow.<br><br>Conclusions<br>GeneSeqToFamily represents the Ensembl Compara pipeline as a set of interconnected Galaxy tools, so they can be run interactively within the Galaxy's user-friendly workflow environment while still providing the flexibility to tailor the analysis by changing configurations and tools if necessary. Additional tools allow users to subsequently visualise the gene families produced by the workflow, using the Aequatus.js interactive tool, which has been developed as part of the Aequatus software project.
**Corresponding Author:**	Anil S Thanki, MSc<br>Earlham Institute<br>Norwich, Norfolk UNITED KINGDOM
**Corresponding Author Secondary Information:**	
**Corresponding Author's Institution:**	Earlham Institute
**Corresponding Author's Secondary Institution:**	
**First Author:**	Anil S Thanki, MSc

Powered by Editorial Manager® and ProduXion Manager® from Aries Systems Corporation

**First Author Secondary Information:**	
**Order of Authors:**	Anil S Thanki, MSc
	Nicola Soranzo, PhD
	Wilfried Haerty, PhD
	Robert P Davey, PhD
**Order of Authors Secondary Information:**	

Response to Reviewers:

>>>Original comment:
>>>First, to test the tool, the objective must be clearer defined. Is the goal to find homologs most generally? Full-length homologs? Orthologs? And if orthologs, then orthologs relative to the set of species used as input or something else? This must be explicitly stated for the evaluation and parameter search to be well-defined. The manuscript should also make clear that this goal is the one that utility for reaching is implied, instead of any other goal.

>>Author response:
>>The main aim of the test is to demonstrate that we are able to identify orthogroups (groups of genes sharing ancestry by descent) across a set of species. In the revised version of the manuscript, we clarified the goals of the tool and its purpose.

>Reviewer response:
>This is still unclear. What paper defined the term "orthogroup" and where do you cite it for this purpose? Where in this MS is that term used? The introduction still does not define gene family, and does not say if these are just homologous or whether they will be orthologous relative to a particular ancestral taxon, and if so which one. The introduction needs a clear definition of gene family, and the method needs to be stated as providing gene families according to this particular definition. Otherwise any data on performance are useless, because it is not possible to say whether or not the stated goals have been reached. Please revise and add this.

Author Response:
We used the same definition of orthogroups as Gabaldón and Koonin (Nature Rev. Genet. 2013), and we have added the citation in the revised version of the manuscript. In addition, we have clarified the introduction by adding the definitions of “gene family” and “homologous genes”. This workflow focuses on the discovery and characterisation of gene families, not specifically on the benchmarking of orthologs or paralogs.

>>>Original comment:
>>>The data set used for parameter searches is tiny, just three species, two of which are obscure (how good are assemblies?). This should be done on a much larger set, perhaps use the Quest for Orthologs' reference dataset?

>>Author response:
>>Although we only used three species, the data set includes over 63,000 genes. The reviewer raises a valid question regarding the quality of the assemblies, i.e. while the mouse genome can be considered of very high quality, both the platypus and Tasmanian devil genome assemblies are of much lower quality. Therefore, we have now run our workflow on two sets of three species, one including high quality assemblies (human, mouse and dog), the second set composed of draft genome assemblies only (pig, platypus and horse). Furthermore, we ran our analysis on all six species to assess the impact of mixed assemblies qualities on the identification of gene families.

>Reviewer response:
>One set of three species, another set of three species, one set of six species - who does this sort of tiny analysis in real practice? Furthermore, just by chance with this few you might perceive trends that would disappear with a more balanced benchmark. This is not sufficiently addressing my concern. Please revise to do so.

Author Response:
Here we chose 3 species with high quality and 3 species with low quality of genome

Powered by Editorial Manager® and ProduXion Manager® from Aries Systems Corporation

assembly to check the impact of quality of a genome on how the workflow is able to discern gene families. Additionally, with the increasing number of genomes released, many being early assemblies, this analysis is of direct interest to the intended users of our workflow, highlighting the impact of assembly quality on gene family detection.
Following the reviewer's comment we also performed an analysis on the 66 species from Quest for Orthologs' (QfO) reference proteome (see below).

>>>Original comment:
>>>Testing six settings does not show that one of the settings consistently give any particular results. The way to do that would be to test each parameter set many times, using different species combinations each time, and check if indeed Analysis 5 parameters consistently yield desired results under a well-defined quality metric (dependent on the goal posts set above).

>>Author response:
>>As per reviewer's request, we tested six sets of parameters on the high quality, low quality, and both high and low quality datasets mentioned above. All datasets tested confirm that Analysis 5 returns the optimal results for these datasets. We acknowledge that different datasets might require further tuning, but we believe these parameters to be suitably comprehensive for most analyses.

>Reviewer response:
>So since my concern 2) was not yet addressed, 3) also was not, since that would require being able to sample the species space more than just 3+3 species allow. I don't understand why what I asked for was not done here? Since this should be an easily usable pipeline for wide audience, doing so should not be so difficult. Please revise.

Author Response:
As per reviewer's request we ran our workflow on QfO reference proteome using various sets of parameters. Due to the size of the results, we provide an additional file alongside our manuscript informing users on the impact of parameter choices.
However, we would like to reiterate that the GeneSeqToFamily workflow is designed to identify gene families, including both 1:1 orthologs and paralogs. During our analysis, we identified two factors that adversely affect Positive Prediction Value Rate achieved using the GeneSeqToFamily pipeline.
Firstly, QfO is designed to assess the specificity and sensitivity of pipelines in calling 1:1 orthologs, whereas the output of our workflow includes gene families with 1:many and many:many orthologs. These 1:many orthologs are counted towards the False Positive (FP) rate by QfO. To validate our findings, we manually checked these FPs against the orthologs listed in the Ensembl Compara database at EBI. We did indeed find that our FPs are listed as 1:many or many:many orthologs in Compara, and therefore we conclude that because these are not recorded as such in the QfO output, there is some data cleaning (most likely tree reconciliation) that is carried out prior to submitting to QfO.
Secondly, because of the phylogenetic diversity of the species used in QfO, it is necessary to provide a category file to run hcluster_sg optimally. Unfortunately neither this file, or the parameters for its compilation are available from Ensembl Compara, so we cannot perform a like-for-like comparison. We are confident that GeneSeqToFamily, based on its comparable true positive rate, and based on the 1:many issue above, is working as expected and of value to the community to assess gene families.

>>>Original comment:
>>>Testing performance on 23 genes is vastly insufficient. Please look into much larger
benchmark sets (e.g. OrthoBench or quest for orthologs' benchmark tool), choose some larger, more diverse (functionally and phylogenetically) set to use as a gold standard, define a metric for success, then show how well the tool works under repeated applications of method settings on different subsets of the data.

>>Author response:
>>We tried running our workflow with 'Quest for orthologs', but the recommended dataset

Powered by Editorial Manager® and ProduXion Manager® from Aries Systems Corporation

available for this tool is very old (2011). We contacted the author of Quest for Orthologs for the latest release (2017), but there is no gold standard resultant data available to compare to, so maybe we can look into benchmarking this workflow in the future when results from other tools are available to compare with.

>Reviewer response:
>This makes no sense. It may be old (2011 is old?) but it is still 60+ chosen species, so much more than you currently use. Furthermore it was specifically designed (with input from specialists) to be such a gold standard, and if it has not changed much, this is because discussions on whether to add/subtract species since have not lead to any consensus that doing so is called for. It is eminently usable, have been so used in other contexts, and works fine for this purpose. If you prefer another set of similar size you can of course use that, but you need to benchmark this on a non-toy dataset. Please revise.

Author Response:
This request should be satisfied by our previous answers.

>>>Original comment:
>>>To be clear, I think this is valuable and worthy of publication, but for any claim of functionality, it must be robustly tested under clear definitions on large-scale diverse and representative data as stated above (which should also have some clear measure of accuracy relative to the gold standard).

>>Author response:
>>We thank the reviewer for their comments, and we hope that the revised manuscript and associated broader analyses will help readers decide whether this workflow is suitable for their datasets. We feel that the mechanisms that the reviewer kindly suggested for assessing our workflow against a gold standard are inadequate to give a representative assessment. We will continue to monitor the available tools and standards in order to provide support for the usefulness of the workflow.

>Reviewer response:
>This is just referencing 5 above. As with that, you need to redo on a properly large benchmark.

Author Response:
As mentioned above, this request should also be satisfied by our previous answers. However, we feel we should comment that the results of QfO are hugely determinant on finding the exact parameters that give the best results for this singular benchmark. Whilst QfO is clearly valuable to inform confidence on already known orthologs, we feel GeneSeqToFamily comprises the best of both worlds - high accuracy (1:many FP issue aside, reported above) and the ability to characterise unknown gene families.

>>>Original comment:
>>> Similarly, please add time and memory consumption statistics for different use cases.

>>Author response:
>>Time and memory consumption for the workflow depends on the infrastructure it is running on. We ran the Galaxy jobs on an High Performance Computing cluster, where measuring the amount of memory used is tricky. Also, the total time used to complete the pipeline may heavily vary depending on the time spent in the cluster scheduler waiting for resources to be available.
Anyhow, most of the time is spent on the NCBI blastp step, which for the examples in the paper varied between 16 and 24 hours for 6 species with 125,342 sequences.

>Reviewer response:
>The cluster should have a queuing system for its nodes, which if anything ought to make assessing memory use easier. 16-24 hours using how many nodes/cores? Please add these numbers to the revised manuscript.

Author Response:
We’ve now collected runtime and memory statistics for a typical run of the

	GeneSeqToFamily workflow (up to the hcluster_sg step, where gene families are determined) on the QfO proteomes.<br>Over 99% of the runtime was spent on BLASTP, which was run in parallel on the 66 protein FASTA files (one for each reference species). The average runtime of BLASTP, using 4 cores, was 735 minutes, with a maximum of 4303 min.<br>The step using the majority of the pipeline memory allocation was hcluster_sg with 2.32 GB.
**Additional Information:**	
**Question**	**Response**
Are you submitting this manuscript to a special series or article collection?	No
**Experimental design and statistics**<br><br>Full details of the experimental design and statistical methods used should be given in the Methods section, as detailed in our Minimum Standards Reporting Checklist. Information essential to interpreting the data presented should be made available in the figure legends.<br><br>Have you included all the information requested in your manuscript?	Yes
**Resources**<br><br>A description of all resources used, including antibodies, cell lines, animals and software tools, with enough information to allow them to be uniquely identified, should be included in the Methods section. Authors are strongly encouraged to cite Research Resource Identifiers (RRIDs) for antibodies, model organisms and tools, where possible.<br><br>Have you included the information requested as detailed in our Minimum Standards Reporting Checklist?	Yes
**Availability of data and materials**<br><br>All datasets and code on which the conclusions of the paper rely must be either included in your submission or deposited in publicly available repositories (where available and ethically appropriate), referencing such data using a unique identifier in the references and in the “Availability of Data and Materials” section of your manuscript.<br><br>Have you have met the above	Yes

requirement as detailed in our Minimum Standards Reporting Checklist?	

Powered by Editorial Manager® and ProduXion Manager® from Aries Systems Corporation

Manuscript Click here to download Manuscript GeneSeqToFamily.docx

GeneSeqToFamily: a Galaxy workflow to find gene families based on the Ensembl Compara GeneTrees

Anil S. Thanki[1], Nicola Soranzo[1], Wilfried Haerty[1], Robert P. Davey[1]

1. Earlham Institute (EI), Norwich Research Park, Norwich NR4 7UZ, UK

ORCID details:
Anil S. Thanki: 0000-0002-8941-444X; Nicola Soranzo: 0000-0003-3627-5340; , Wilfried Haerty: 0000-0003-0111-191X; Robert P. Davey: 0000-0002-5589-7754.

Abstract

Background

Gene duplication is a major factor contributing to evolutionary novelty, and the contraction or expansion of gene families has often been associated with morphological, physiological and environmental adaptations. The study of homologous genes helps us to understand the evolution of gene families. It plays a vital role in finding ancestral gene duplication events as well as identifying genes that have diverged from a common ancestor under positive selection. There are various tools available, such as MSOAR, OrthoMCL and HomoloGene, to identify gene families and visualise syntenic information between species, providing an overview of syntenic regions evolution at the family level. Unfortunately, none of them provide information about structural changes within genes, such as the conservation of ancestral exon boundaries amongst multiple genomes. The Ensembl GeneTrees computational pipeline generates gene trees based on coding sequences and provides details about exon conservation, and is used in the Ensembl Compara project to discover gene families.

Findings

A certain amount of expertise is required to configure and run the Ensembl Compara GeneTrees pipeline via command line. Therefore, we have converted the command line Ensembl Compara GeneTrees pipeline into a Galaxy workflow, called GeneSeqToFamily, and provided additional functionality. This workflow uses existing tools from the Galaxy ToolShed, as well as providing additional wrappers and tools that are required to run the workflow.

Conclusions

GeneSeqToFamily represents the Ensembl Compara pipeline as a set of interconnected Galaxy tools, so they can be run interactively within the Galaxy's user-friendly workflow environment while still providing the flexibility to tailor the analysis by changing configurations and tools if necessary. Additional tools allow users to subsequently visualise the gene families produced by

the workflow, using the Aequatus.js interactive tool, which has been developed as part of the Aequatus software project.

Keywords

Galaxy, Pipeline, Workflow, Genomics, Comparative Genomics, Homology, Orthology, Paralogy, Phylogeny, Gene Family, Alignment, Compara, Ensembl

Introduction

The phylogenetic information inferred from the study of homologous genes helps us to understand the evolution of gene families (also referred to as “orthogroups”), that comprise genes sharing common descent [1]. This plays a vital role in finding ancestral gene duplication events as well as identifying regions under positive selection within species [2]. In order to investigate these low-level comparisons between gene families, the Ensembl Compara GeneTrees gene orthology and paralogy prediction software suite [3] was developed as a pipeline. The Ensembl pipeline uses TreeBest [4][5] (part of TreeFam [6]), which implements multiple independent phylogenetic methods and can merge the results into a consensus tree whilst trying to minimise duplications and deletions relative to a known species tree. This allows TreeBeST to take advantage of the fact that DNA-based methods are often more accurate for closely related parts of trees, while protein-based trees are better at longer evolutionary distances.

The Ensembl GeneTrees pipeline comprises seven steps, starting from a set of protein sequences and performing similarity searching and multiple large-scale alignments to infer homology among them, using various tools: BLAST [7], hcluster_sg [8], T-Coffee [9], and phylogenetic tree construction tools, including TreeBeST. Whilst these tools are freely available, most are specific to certain computing environments, are only usable via the command line, and require many dependencies to be fulfilled. Therefore, users are not always sufficiently expert in system administration in order to install, run, and debug the various tools at each stage in a chain of processes. To help ease the complexity of running the GeneTrees pipeline, we have employed the Galaxy bioinformatics analysis platform to relieve the burden of managing these system-level challenges.

Galaxy is an open-source framework for running a broad collection of bioinformatics tools via a user-friendly web interface [10]. No client software is required other than a recent web browser, and users are able to run tools singly or aggregated into interconnected pipelines, called *workflows*. Galaxy enables users to not only create, but also share workflows with the community. In this way, it helps users who have little or no bioinformatics expertise to run potentially complex pipelines in order to analyse their own data and interrogate results within a single online platform. Furthermore, pipelines can be published in a scientific paper or in a repository such as myExperiment [11] to encourage transparency and reproducibility.

In addition to analytical tools, Galaxy also contains plugins [12] for data visualisation. Galaxy visualisation plugins may be interactive and can be configured to visualise various data types, for example, bar plots, scatter plots, and phylogenetic trees. It is also possible to develop custom visualisation plugins and easily integrate them into Galaxy. As the output of the GeneSeqToFamily workflow is not conducive to human readability, we also provide a data-to-visualisation plugin based on the Aequatus software [13]. Aequatus.js [14] is a new JavaScript library for the visualisation of homologous genes, which is extracted from the standalone Aequatus tool. It provides a detailed view of gene structure across gene families, including shared exon information within gene families alongside gene tree representations. It also shows details about the type of interrelation event that gave rise to the family, such as speciation, duplication, and gene splits.

Methods

The GeneSeqToFamily workflow has been developed to run the Ensembl Compara software suite within the Galaxy environment (Galaxy , RRID:SCR_006281), combining various tools alongside preconfigured parameters obtained from the Ensembl Compara pipeline to produce gene trees. Among the tools used in GeneSeqToFamily (listed in Table 1), some were existing tools in the Galaxy ToolShed [15], such as NCBI BLAST (NCBI BLAST , RRID:SCR_004870), TranSeq (Transeq, RRID:SCR_015647), Tranalign and various format converters. Additional tools that are part of the pipeline were developed at the Earlham Institute (EI) and submitted to the ToolShed, i.e. *BLAST parser, hcluster_sg, hcluster_sg parser, T-Coffee, TreeBeST best* and *Gene Alignment and Family Aggregator.* Finally, we developed helper tools that are not part of the workflow itself, but aid the generation of input data for the workflow and these are also in the ToolShed, i.e. *Get features by Ensembl ID*, *Get sequences by Ensembl ID*, *Select longest CDS per gene*, *ETE species tree generator* and *GeneSeqToFamily preparation*.

The workflow comprises 7 main steps, starting with translation from input coding sequences (CDS) to protein sequences, finding subsequent pairwise alignments of those protein sequences using BLASTP, and then the generation of clusters from the alignments using hcluster_sg. The workflow then splits into two simultaneous paths, whereby in one path it performs the multiple sequence alignment (MSA) for each cluster using T-Coffee (T-Coffee,

Figure 1: Overview of the GeneSeqToFamily workflow

RRID:SCR_011818), and in the other, generates a gene tree with TreeBeST taking the cluster alignment and a species tree as input. Finally, these paths merge to aggregate the MSA, the gene tree and the gene feature information (transcripts, exons, and so on) into an SQLite [16] database for visualisation and downstream reuse. Each step of the workflow along with the data preparation steps is explained in detail below.

Figure 2: Screenshot from the Galaxy Workflow Editor, showing the GeneSeqToFamily workflow

Table 1: Galaxy tools used in the workflow

Tool Name	Tool ID	Version	Developed at EI		ToolShed Reference
			Tool	Wrapper	
Get sequences by Ensembl ID	get_sequences	0.1.2	Yes	Yes	[17]
Get features by Ensembl ID	get_feature_info	0.1.2	Yes	Yes	[18]
Select longest CDS per gene	ensembl_longest_cds_per_gene	0.0.2	Yes	Yes	[19]
ETE species tree generator	ete_species_tree_generator	3.0.0b35	Yes	Yes	[20]
GeneSeqToFamily preparation	gstf_preparation	0.4.0	Yes	Yes	[21]
Transeq	EMBOSS: transeq101	5.0.0	No	No	[22]
NCBI BLAST+ makeblastdb	ncbi_makeblastdb	0.2.01	No	No	[23]
NCBI BLAST+ blastp	ncbi_blastp_wrapper	0.2.01	No	No	[23]
BLAST parser	blast_parser	0.1.2	Yes	Yes	[24]
hcluster_sg	hcluster_sg	0.5.1.1	No	Yes	[25]
hcluster_sg parser	hcluster_sg_parser	0.2.0	Yes	Yes	[26]
Filter by FASTA IDs	filter_by_fasta_ids	1.0	No	No	[27]
T-Coffee	t_coffee	11.0.8	No	Yes	[28]
Tranalign	EMBOSS: tranalign100	5.0.0	No	No	[22]
TreeBeST best	treebest_best	1.9.2	No	Yes	[29]
Gene Alignment and Family Aggregator	gafa	0.3.0	Yes	Yes	[30]
Unique	tp_sorted_uniq	1.1.0	No	No	[31]
FASTA-to-Tabular	fasta2tab	1.1.0	No	No	[32]
UniProt ID mapping and retrieval	uniprot_rest_interface	0.1	No	No	[33]

Data generation and preparation

We have developed a number of tools that assist in preparing the datasets needed by the workflows.

Ensembl REST API tools

Galaxy tools were developed which utilise the Ensembl REST API [34] to retrieve sequence information (*Get sequences by Ensembl ID*) and feature information (*Get features by Ensembl ID*) by Ensembl ID from the Ensembl service. REST (REpresentational State Transfer) is an architecture style for designing networked applications [35] which encourages the use of standardised HTTP technology to send and receive data between computers. As such, these tools are designed to help users to retrieve existing data from Ensembl rather than requiring them to manually download datasets to their own computers and then subsequently uploading them into the workflow.

We have also developed the

- *ETE species tree generator* Galaxy tool, which uses the ETE toolkit [36] to generate a species tree from a list of species names or taxon IDs through the NCBI Taxonomy.

GeneSeqToFamily workflow

0. GeneSeqToFamily preparation

Before GeneSeqToFamily can be run, a data preparation step must be carried out. We have developed a tool called *GeneSeqToFamily preparation* to preprocess the input datasets (gene feature information and CDS) for the GeneSeqToFamily workflow. It converts a set of gene feature information files in GFF3 [37] and/or JavaScript Object Notation (JSON) [38] format to an SQLite database. It also modifies all CDS FASTA header lines by appending the species name to the transcript identifier, as required by *TreeBeST best*. It can also retain only the longest CDS sequence for each gene, as done in the Compara pipeline.

We decided to use an SQLite database to store the gene feature information because:

- the GFF3 format has a relatively inconvenient and unstructured additional information field (9th column)
- searching is much faster and more memory efficient in a database than in a text file like JSON or GFF3, especially when dealing with feature information for multiple large genomes

1. CDS translation

Transeq

Transeq, part of the European Molecular Biology Open Software Suite (EMBOSS) (EMBOSS, RRID:SCR_008493)[39], is a tool to generate six-frame translation of nucleic acid sequences to their corresponding peptide sequences. Here we use Transeq to convert a CDS to protein sequences in order to run BLASTP (BLASTP , RRID:SCR_001010) and find protein clusters.

However, since downstream tools in the pipeline such as TreeBeST require nucleotide sequences to generate a gene tree, the protein sequences cannot be directly used as workflow input and are instead generated with Transeq.

2. Pre-clustering alignment

BLAST

This workflow uses the BLAST wrappers [40] developed to run BLAST+ tools within Galaxy. BLASTP is run over the set of sequences against the database of the same input, as is the case with BLAST-all, in order to form clusters of related sequences.

BLAST parser

BLAST parser is a small Galaxy tool to convert the BLAST output into the input format required by hcluster_sg. It takes the BLAST 12-column output [41] as input and generates a 3-column tabular file, comprising the BLAST query, the hit result, and the edge weight. The weight value is simply calculated as minus $\log_{10}$ of the BLAST e-value divided by 2, replacing this with 100 if this value is greater than 100. It also removes the self-matching BLAST results and let the user filter out non Reciprocal Best Hits (RBH).

3. Cluster generation

hcluster_sg

hcluster_sg performs clustering for sparse graphs. It reads an input file that describes the similarity between two sequences, and iterates through the process of grouping two nearest nodes at each iteration. hcluster_sg outputs a single list of gene clusters, each comprising a set of sequence IDs present in that cluster. This list needs to be reformatted using the *hcluster_sg parser* tool in order to be suitable for input into T-Coffee and TreeBeST (see below).

hcluster_sg parser

hcluster_sg parser converts the hcluster_sg output into a collection of lists of IDs, one list for each cluster. Each of these clusters will then be used to generate a gene tree via TreeBeST. The tool can also filter out clusters with a number of elements outside a specified range. The IDs contained in all discarded clusters are collected in separate output dataset. Since TreeBeST requires at least 3 genes to generate a gene tree, we configured the tool to filter out clusters with less than 3 genes.

Filter by FASTA IDs, which is available from the Galaxy ToolShed, is used to create separate FASTA files using the sequence IDs listed in each gene cluster.

4. Cluster alignment

T-Coffee

T-Coffee is a MSA package, but can also be used to combine the output of other alignment methods (Clustal, MAFFT, Probcons, MUSCLE) into a single alignment. T-Coffee can align both nucleotide and protein sequences [9], and we use it to align the protein sequences in each cluster generated by hcluster_sg.

We modified the Galaxy wrapper for T-Coffee to take a single FASTA (as normal) and an optional list of FASTA IDs to filter. If a list of IDs is provided, the wrapper will pass only those sequences to T-Coffee, which will perform the MSA for that set of sequences, thus removing the need to create thousands of intermediate Galaxy datasets.

5. Gene tree construction

Tranalign

Tranalign [39] is a tool that reads a set of nucleotide sequences and a corresponding aligned set of protein sequences and returns a set of aligned nucleotide sequences. Here we use it to generate CDS alignments of gene sequences using the protein alignments produced by T-Coffee.

TreeBeST 'best'

TreeBeST (Tree Building guided by Species Tree) is a tool to generate, manipulate, and display phylogenetic trees and can be used to build gene trees based on a known species tree.

The 'best' command of TreeBeST builds 5 different gene trees from a FASTA alignment file using different phylogenetic algorithms, then merges them into a single consensus tree using a species tree as a reference. In GeneSeqToFamily, *TreeBeST best* uses the nucleotide MSAs generated by Tranalign (at least 3 sequences are required) and a user-supplied species tree in Newick format [42] (either produced by a third-party software or through the *ETE species tree generator* data preparation tool, described above) to produce a GeneTree for each family represented also in Newick format. The resulting GeneTree also includes useful annotations specifying phylogenetic information of events responsible for the presence/absence of genes, for example, 'S' means speciation event, 'D' means duplication, and 'DCS' denotes the duplication score.

6. Gene Alignment and Family Aggregation

Gene Alignment and Family Aggregator (GAFA)

GAFA is a Galaxy tool which generates a single SQLite database containing the gene trees and MSAs, along with gene features, in order to provide a reusable, persistent data store for visualisation of synteny information with Aequatus. *GAFA* requires:

- gene trees in Newick format,
- the protein MSAs in fasta_aln format from *T-Coffee* and
- gene feature information generated with the *GeneSeqToFamily preparation* tool.

Internally, *GAFA* converts each MSA from fasta_aln format to a simple CIGAR string [43]. An example of CIGAR strings for aligned sequences is shown in Figure 3, in which each CIGAR string subset changes according to other sequences.
The simple schema [44] for the generated SQLite database is shown in Figure 4.

Figure 3: Showing how CIGAR for multiple sequence alignment is generated

Figure 4: Schema of the GAFA SQLite database

7. Visualisation

Aequatus visualisation plugin

The SQLite database generated by the GAFA tool can be rendered using a new visualisation plugin, Aequatus.js. The Aequatus.js library, developed at EI as part of the Aequatus project, has been configured to be used within Galaxy to visualise homologous gene structure and gene family relationships. This allows users to interrogate not only the evolutionary history of the gene family but also the structural variation (exon gain/loss) within genes across the phylogeny. Aequatus.js is available to download from GitHub [44], as visualisation plugins cannot yet be submitted to the Galaxy ToolShed.

Finding homology information for orphan genes

Although the GeneSeqToFamily workflow will assign most of the genes to orthogroups, many genes within a species might appear to be unique without homologous relationship to any other genes from other species. This observation could be the consequence of the parameters selected, choice of species or incomplete annotations. This could also reflect real absence of homology such as for rapidly evolving gene families. In addition to the GeneSeqToFamily workflow, we also developed two associated workflows to further annotate these genes by:

1) Retrieving a list of orphan genes from the GeneSeqToFamily workflow (see Figure 5) as follows:
    a) Find the IDs of the sequences present in the input CDS of the GeneSeqToFamily workflow, but not in the result of *BLAST parser* from the same workflow
    b) Add to this list the IDs of the sequences discarded by *hcluster_sg parser*
    c) From the input CDS dataset, retrieve the respective sequence for each CDS ID (from the step above) using *Filter by FASTA IDs*

    These unique CDS can be fed into the SwissProt workflow below to find homologous genes in other species.
2) Finding homologous genes for some genes of interest using SwissProt (see Figure 6) as follows:
    a) Translate CDS into protein sequences using *Transeq*
    b) Run BLASTP for the protein sequences against the SwissProt database (from NCBI)
    c) Extract UniProt IDs from these BLASTP results, using the preinstalled Galaxy tool *Cut columns from a table* (tool id *Cut1*)
    d) Retrieve Ensembl IDs (representing genes and/or transcripts) for each UniProt ID using *UniProt ID mapping and retrieval*
    e) Get genomic information for each gene ID and CDS for each transcript ID from the core Ensembl database using *Get features by Ensembl ID* and *Get sequences by Ensembl ID* respectively.

    The results from this second workflow can be subsequently used as input to GeneSeqToFamily for familial analysis.

Figure 5: Screenshot from the Galaxy Workflow Editor, showing the orphan gene finding workflow

Figure 6: Screenshot from the Galaxy Workflow Editor, showing the SwissProt workflow

Results

To validate the biological relevance of results from the GeneSeqToFamily workflow, we analysed a small set of 23 homologous genes (1 transcript per gene) from *Pan troglodytes* (chimpanzee), *Homo sapiens* (human), *Rattus norvegicus* (rat), *Mus musculus* (mouse), *Sus scrofa* (pig) and *Canis familiaris* (domesticated dog). These genes are a combination of those found in three gene families, i.e. monoamine oxidases (MAO A and B), insulin receptor (INSR), BRCA2, and were chosen because they are present in all 6 species yet distinct from each other. Before running the workflow, feature information and CDS for the selected genes were retrieved from the core Ensembl database using the helper tools described above (*Get features by Ensembl ID* and *Get sequences by Ensembl ID* respectively) and CDS were filtered to keep longest CDS per gene. A species tree was generated using *ETE species tree generator* and CDS were prepared with *GeneSeqToFamily preparation*.

We ran the GeneSeqToFamily workflow on these data using the default parameters of the Ensembl Compara pipeline (Table 2, experiment D). This workflow generated 3 different gene trees, each matching exactly one gene family. Figure 7, 8 and 9 show the resulting gene trees for MAO, BRCA2 and INSR gene families. Different colours of the nodes in each gene tree on the left-hand-side of the visualisation highlight potential evolutionary events, such as speciation, duplication, and gene splits. Homologous genes showing shared exons use the same colour in each representation, including insertions (black blocks) and deletions (red lines). The GeneTrees for these genes are already available in Ensembl and we used them to validate our findings [45] [46] [47] [48]. Our gene trees agree perfectly with the Ensembl GeneTrees, showing that the workflow generates biologically valid results. We have provided the underlying data for this example along with the submitted workflow in figshare [49].

Figure 7: Homologous genes of MAO of *Canis familiaris* from *Mus musculus, Pan troglodytes, Homo sapiens, Rattus norvegicus, Sus scrofa and Canis familiaris*.

Figure 8: Homologous genes of BRCA2 of *Canis familiaris* from *Mus musculus, Pan troglodytes, Homo sapiens, Rattus norvegicus, Sus scrofa and Canis familiaris*.

Figure 9: Homologous genes of INSR of *Mus musculus* from *Pan troglodytes, Homo sapiens, Rattus norvegicus, Sus scrofa and Canis familiaris*.

We also studied the impact of the most important tool parameters on the gene families reconstructed by the workflow by running it on larger datasets, in particular the reference proteomes of 754,149 sequences from 66 species established by the Quest for Orthologs (QfO) consortium [50]. We ran GeneSeqToFamily (up to the hcluster_sg step, where gene families are

determined) with various sets of parameters (shown in Table 2) and performed statistical analysis on the resulting gene families (Table 3). Our results show that the number of gene families can vary quite distinctly with different BLASTP and hcluster_sg parameters. Stringent parameters (Parameter Set F) result in a large number of smaller families, while relaxed parameters (Parameter Set A) generate a small number of larger families, which may include distantly related genes. The parameters used by Ensembl Compara as default are shown in Parameter Set D.

Table 2: Set of parameters used in BLASTP and hcluster_sg to compare results. BLASTP was configured with maximum number of HSPs set to 1, and hcluster_sg with single link clusters set to 'no' and maximum size set to 500.

Tool	Parameter	Parameter set					
		A	B	C	D	E	F
BLASTP	expectation value cutoff	1e-03	1e-03	1e-03	1e-10	1e-10	1e-10
	Query coverage per hsp	0	0	90	0	0	90
hcluster_sg	Minimum edge weight	0	20	0	0	20	20
	Minimum edge density between a join	0.34	0.50	0.34	0.34	0.50	0.50

Table 3: Results of the GeneSeqToFamily workflow run with 7 different set of parameters, the complete list of which are shown in Table 2.

Summary						
**Analysis**	**A**	**B**	**C**	**D**	**E**	**F**
**No of genes**	754,149	754,149	754,149	754,149	754,149	754,149
**No of families**	58,272	74,252	83,900	63,289	74,309	79,879
**No of larger families (>200)**	435	168	56	350	167	46
**No of smaller families (<3)**	30,563	40,530	44,295	33,308	40,579	41,794
**Families (>3 and <200)**	27,274	33,556	39,548	29,628	33,562	38,039
**Largest family size**	615	567	556	652	561	527
**Average family size**	11.38	7.36	5.36	10.04	7.35	5.09

We also performed benchmarking using the QfO benchmarking service [50]. QfO benchmarking focuses on assessing the accuracy of a tool to predict 1:1 orthology, whilst the GeneSeqToFamily workflow focuses on whole gene families, regardless of the type of homology among the members of a gene family. GeneSeqToFamily performs comparably to other tools benchmarked in QfO, even surpassing them for True Positive ortholog discovery in some parameter spaces. However, we found issues with the QfO service recording 1:many orthologs as False Positives, hence reducing our overall specificity. Additional information about the corresponding results of benchmarking are available in Additional File 1.

Conclusion

The ultimate goal of the GeneSeqToFamily is to provide a user-friendly workflow to analyse and discover homologous genes and their corresponding gene families using the Ensembl Compara GeneTrees pipeline within the Galaxy framework, where users can interrogate genes of interest without using the command-line whilst still providing the flexibility to tailor analysis by changing configurations and tools if necessary. We have shown it to be an accurate, robust, and reusable method to elucidate and analyse potentially large numbers of gene families in a range of model and non-model organisms. The workflow stores the resulting gene families into a SQLite database, which can be visualised using the Aequatus.js interactive tool, as well as shared as a complete reproducible container for potentially large gene family datasets.

We invite the Galaxy community to undertake their own analyses and feedback improvements to various tools, and publish successful combinations of parameters used in the GeneSeqToFamily workflow to achieve better gene families for their datasets. We encourage this process by allowing users to share their own version of GeneSeqToFamily workflow for appraisal by the community.

Future directions

In terms of core workflow functionality, we would like to incorporate pairwise alignment between pairs of genes for closely related species in addition of the MSA for the gene family, which will help users to compare orthologs and paralogs in greater detail.

We also plan to explicitly include the PantherDB resources [51]. Protein ANalysis THrough Evolutionary Relationships (PANTHER) is a classification system to characterise known proteins and genes according to family, molecular function, biological process and pathway. The integration of PantherDB with GeneSeqToFamily will enable the automation of gene family validation and add supplementary information about those gene families, which could in turn be used to further validate novel genomics annotation.

Finally we intend to add the ability to query the *GAFA* SQLite database using keywords, to make it easy for users to find gene trees which include their genes of interest without needing to delve into the database itself.

Availability and requirements

Project name: GeneSeqToFamily
Project home page: https://github.com/TGAC/earlham-galaxytools/tree/master/workflows/GeneSeqToFamily
Archived version: 0.1.0
Operating system(s): Platform independent
Programming language: JavaScript, Perl, Python, XML, SQL
Other Requirements: Web Browser; for development: Galaxy
Any restrictions to use by non-academics: None
License: The MIT License (https://opensource.org/licenses/MIT)

Availability of supporting data

The example files and additional data sets supporting the results of this article are available in figshare [49]. A virtual image for Galaxy with necessary tools and installed workflow also available at Earlham repos [52]. Snapshots of the supporting data and code are also hosted in the *GigaScience* GigaDB repository [53].

Acknowledgements

AT, WH and RPD are supported by BBSRC institute strategic programme grant funds awarded to EI. NS is funded under the BBSRC Biomathematics and Bioinformatics Training fund (2014) awarded to EI. This research was supported in part by the NBI Computing infrastructure for Science (CiS) group who provide technical support and maintenance to EI's High Performance Computing cluster and storage systems, enabling us to develop this workflow.

We would like to thank Matthieu Muffato from the European Bioinformatics Institute (EBI) for his advice during the initial stage of the project.

References

1. Gabaldón T, Koonin EV. Functional and evolutionary implications of gene orthology. Nat Rev Genet. 2013;14:360–6.

2. Jensen JD, Wong A, Aquadro CF. Approaches for identifying targets of positive selection. Trends Genet. 2007;23:568–77.

3. Vilella AJ, Severin J, Ureta-Vidal A, Heng L, Durbin R, Birney E. EnsemblCompara GeneTrees: Complete, duplication-aware phylogenetic trees in vertebrates. Genome Res.

2008;19:327–35.

4. Ensembl. Ensembl/treebest. GitHub. https://github.com/Ensembl/treebest. Accessed 26 Jan 2016.

5. Heng L. Constructing the TreeFam database. The Institute of Theoretical Physics, Chinese Academic of Science; 2006. http://pfigshare-u-files.s3.amazonaws.com/1421613/PhDthesisliheng2006English.pdf.

6. Ruan J, Li H, Chen Z, Coghlan A, Coin LJM, Guo Y, et al. TreeFam: 2008 Update. Nucleic Acids Res. 2008;36 Database issue:D735–40.

7. Altschul SF, Gish W, Miller W, Myers EW, Lipman DJ. Basic local alignment search tool. J Mol Biol. 1990;215:403–10.

8. Li H et al. hcluster_sg: hierarchical clustering software for sparse graphs. https://github.com/douglasgscofield/hcluster. Accessed 26 Jan 2016.

9. Notredame C, Higgins DG, Heringa J. T-Coffee: A novel method for fast and accurate multiple sequence alignment. J Mol Biol. 2000;302:205–17.

10. Afgan E, Baker D, van den Beek M, Blankenberg D, Bouvier D, Čech M, et al. The Galaxy platform for accessible, reproducible and collaborative biomedical analyses: 2016 update. Nucleic Acids Res. 2016;44:W3–10.

11. Goble CA, Bhagat J, Aleksejevs S, Cruickshank D, Michaelides D, Newman D, et al. myExperiment: a repository and social network for the sharing of bioinformatics workflows. Nucleic Acids Res. 2010;38 Web Server issue:W677–82.

12. Goecks J, Eberhard C, Too T, Galaxy Team, Nekrutenko A, Taylor J. Web-based visual analysis for high-throughput genomics. BMC Genomics. 2013;14:397.

13. Thanki AS, Ayling S, Herrero J, Davey RP. Aequatus: An open-source homology browser. bioRxiv. 2016;:055632. doi:10.1101/055632.

14. TGAC. TGAC/aequatus.js. GitHub. https://github.com/TGAC/aequatus.js. Accessed 26 Jan 2016.

15. Blankenberg D, Von Kuster G, Bouvier E, Baker D, Afgan E, Stoler N, et al. Dissemination of scientific software with Galaxy ToolShed. Genome Biol. 2014;15:403.

16. SQLite Home Page. https://www.sqlite.org/. Accessed 18 Nov 2016.

17. Get sequences by Ensembl ID : Galaxy Tool Shed. https://toolshed.g2.bx.psu.edu/view/earlhaminst/ensembl_get_sequences/. Accessed 20 Dec 2016.

18. Get features by Ensembl ID : Galaxy Tool Shed. https://toolshed.g2.bx.psu.edu/view/earlhaminst/ensembl_get_feature_info/. Accessed 20 Dec 2016.

19. Select longest CDS per gene : Galaxy Tool Shed. https://toolshed.g2.bx.psu.edu/view/earlhaminst/ensembl_longest_cds_per_gene/. Accessed 8

Mar 2017.

20. ETE species tree generator : Galaxy Tool Shed. https://toolshed.g2.bx.psu.edu/view/earlhaminst/ete/. Accessed 20 Dec 2016.

21. GeneSeqToFamily preparation : Galaxy Tool Shed. https://toolshed.g2.bx.psu.edu/view/earlhaminst/gstf_preparation/. Accessed 17 Mar 2017.

22. EMBOSS : Galaxy Tool Shed. https://toolshed.g2.bx.psu.edu/view/devteam/emboss_5/. Accessed 21 Dec 2016.

23. NCBI BLAST plus : Galaxy Tool Shed. https://toolshed.g2.bx.psu.edu/view/devteam/ncbi_blast_plus. Accessed 21 Dec 2016.

24. BLAST parser : Galaxy Tool Shed. https://toolshed.g2.bx.psu.edu/view/earlhaminst/blast_parser/. Accessed 20 Dec 2016.

25. hcluster_sg : Galaxy Tool Shed. https://toolshed.g2.bx.psu.edu/view/earlhaminst/hcluster_sg/. Accessed 20 Dec 2016.

26. hcluster_sg parser : Galaxy Tool Shed. https://toolshed.g2.bx.psu.edu/view/earlhaminst/hcluster_sg_parser/. Accessed 20 Dec 2016.

27. Filter by FASTA IDs : Galaxy Tool Shed. https://toolshed.g2.bx.psu.edu/view/galaxyp/filter_by_fasta_ids/. Accessed 21 Dec 2016.

28. T-Coffee : Galaxy Tool Shed. https://toolshed.g2.bx.psu.edu/view/earlhaminst/t_coffee/. Accessed 20 Dec 2016.

29. TreeBeST best : Galaxy Tool Shed. https://toolshed.g2.bx.psu.edu/view/earlhaminst/treebest_best/. Accessed 20 Dec 2016.

30. Gene Align and Family Aggregator (GAFA) : Galaxy Tool Shed. https://toolshed.g2.bx.psu.edu/view/earlhaminst/gafa/. Accessed 21 Dec 2016.

31. text_processing : Galaxy Tool Shed. https://toolshed.g2.bx.psu.edu/view/bgruening/text_processing/. Accessed 19 Apr 2017.

32. FASTA-to-Tabular converter : Galaxy Tool Shed. https://toolshed.g2.bx.psu.edu/view/devteam/fasta_to_tabular/. Accessed 19 Apr 2017.

33. uniprot_rest_interface : Galaxy Tool Shed. https://toolshed.g2.bx.psu.edu/view/bgruening/uniprot_rest_interface/. Accessed 20 Mar 2017.

34. Yates A, Beal K, Keenan S, McLaren W, Pignatelli M, Ritchie GRS, et al. The Ensembl REST API: Ensembl Data for Any Language. Bioinformatics. 2015;31:143–5.

35. Representational State Transfer. http://www.peej.co.uk/articles/rest.html. Accessed 4 Feb 2016.

36. Huerta-Cepas J, Serra F, Bork P. ETE 3: Reconstruction, Analysis, and Visualization of Phylogenomic Data. Mol Biol Evol. 2016. doi:10.1093/molbev/msw046.

37. GFF3 - GMOD. http://gmod.org/wiki/GFF3. Accessed 4 Feb 2016.

38. JSON. http://www.json.org. Accessed 4 Feb 2016.

39. Rice P, Longden I, Bleasby A. EMBOSS: the European Molecular Biology Open Software Suite. Trends Genet. 2000;16:276–7.

40. Cock PJA, Chilton JM, Grüning B, Johnson JE, Soranzo N. NCBI BLAST+ integrated into Galaxy. Gigascience. 2015;4:39.

41. National Center for Biotechnology Information (U.S.), Camacho C. BLAST(r) Command Line Applications User Manual. 2008.

42. "Newick's 8:45" Tree Format Standard. http://evolution.genetics.washington.edu/phylip/newick_doc.html. Accessed 8 Apr 2016.

43. Sequence Alignment/Map Format Specification. http://samtools.github.io/hts-specs/SAMv1.pdf. Accessed 20 Dec 2016.

44. TGAC. TGAC/earlham-galaxytools. GitHub. https://github.com/TGAC/earlham-galaxytools. Accessed 21 Mar 2016.

45. Gene: BRAT1 (ENSG00000106009) - Gene tree - Homo sapiens - Ensembl genome browser 87. http://dec2016.archive.ensembl.org/Homo_sapiens/Gene/Compara_Tree?g=ENSG00000106009;r=7:2537877-2555727; Accessed 23 Dec 2016.

46. Gene: INSR (ENSG00000171105) - Gene tree - Homo sapiens - Ensembl genome browser 87. http://dec2016.archive.ensembl.org/Homo_sapiens/Gene/Compara_Tree?g=ENSG00000171105;r=19:7112255-7294034; Accessed 23 Dec 2016.

47. Gene: MAOA (ENSG00000189221) - Gene tree - Homo sapiens - Ensembl genome browser 87. http://dec2016.archive.ensembl.org/Homo_sapiens/Gene/Compara_Tree?g=ENSG00000189221;r=X:43654907-43746824; Accessed 23 Dec 2016.

48. Gene: MAOB (ENSG00000069535) - Gene tree - Homo sapiens - Ensembl genome browser 87. http://dec2016.archive.ensembl.org/Homo_sapiens/Gene/Compara_Tree?g=ENSG00000069535;r=X:43766611-43882447; Accessed 23 Dec 2016.

49. Thanki AS, Soranzo N, Haerty W, Davey R. GeneSeqToFamily.zip. 2017. doi:10.6084/m9.figshare.4484141.v15.

50. Kuzniar A, van Ham RCHJ, Pongor S, Leunissen JAM. The quest for orthologs: finding the corresponding gene across genomes. Trends Genet. 2008;24:539–51.

51. Mi H, Lazareva-Ulitsky B, Loo R, Kejariwal A, Vandergriff J, Rabkin S, et al. The PANTHER database of protein families, subfamilies, functions and pathways. Nucleic Acids Res. 2005;33 Database issue:D284–8.

52. Galaxy Virtual Image. http://repos.tgac.ac.uk/vms/Galaxy_with_GeneSeqToFamily.ova.

Accessed 28 Jul 2017.

53. Thanki, A, S; Soranzo, N; Haerty, W; Davey, R, P (2018): Supporting data for "GeneSeqToFamily: a Galaxy workflow to find gene families based on the Ensembl Compara GeneTrees" GigaScience Database. http://dx.doi.org/10.5524/100402

Click here to download Figure 1.JPG

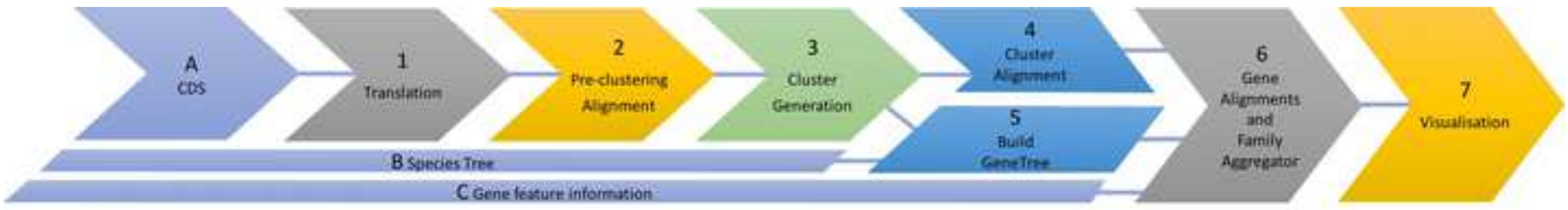

Click here to download Figure 2.png

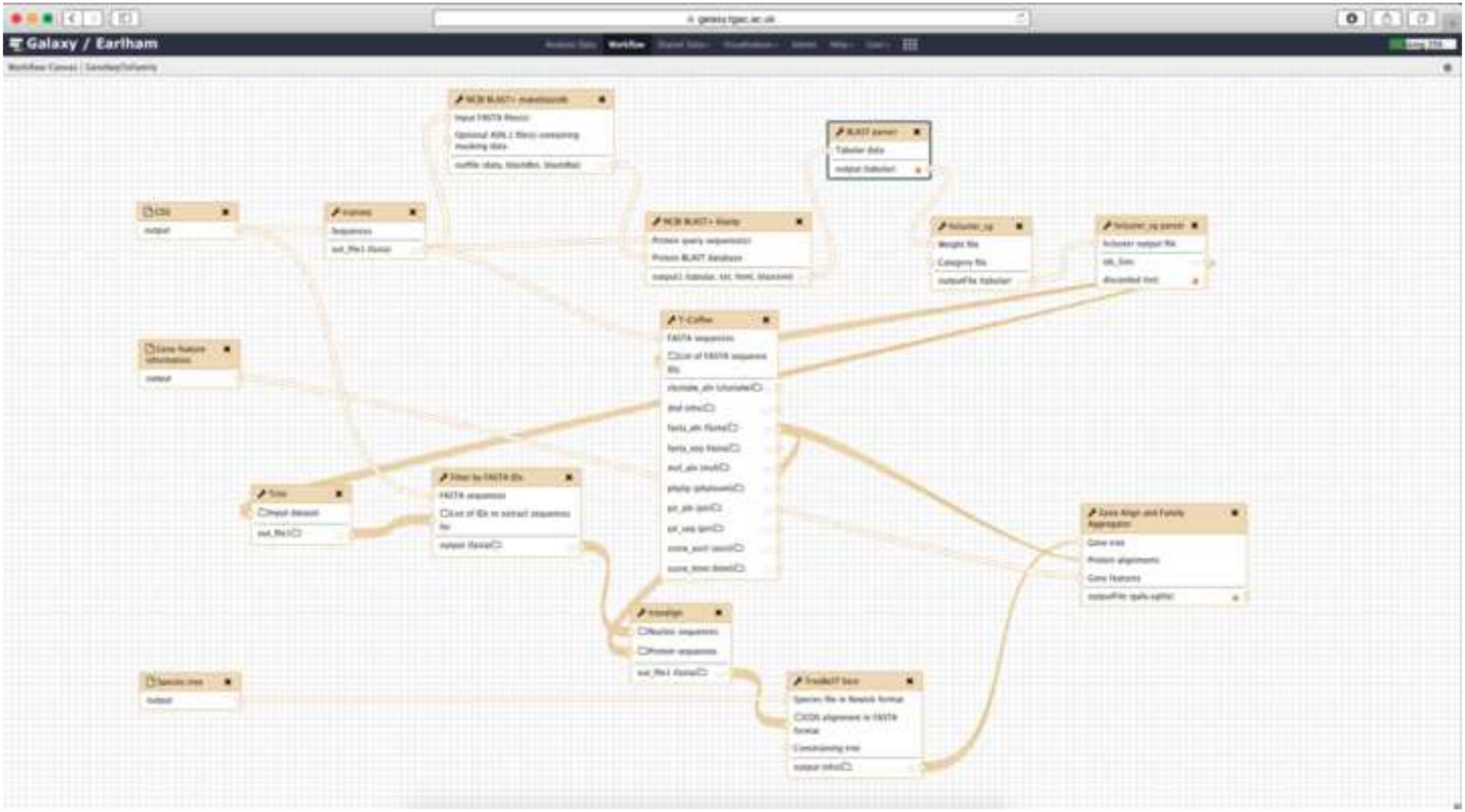

Click here to download Figure 3.png

Sequence1: NLYIQWLKDGGPSSGRPPPS
Sequence2: NLYIQWLKDQGPSSGRPPPS
Sequence3: GDAYAQWLADGGPSSGRPPPSG

Sequence1: -NLYIQWLKDGGPSSGRPPP-S
Sequence2: -NLYIQWLKDQGPSSGRPPP-S
Sequence3: GDAYAQWLADGGPSSGRPPPSG

CIGAR1: D19MDM
CIGAR2: D19MDM
CIGAR3: 22M

Click here to download Figure 4.png

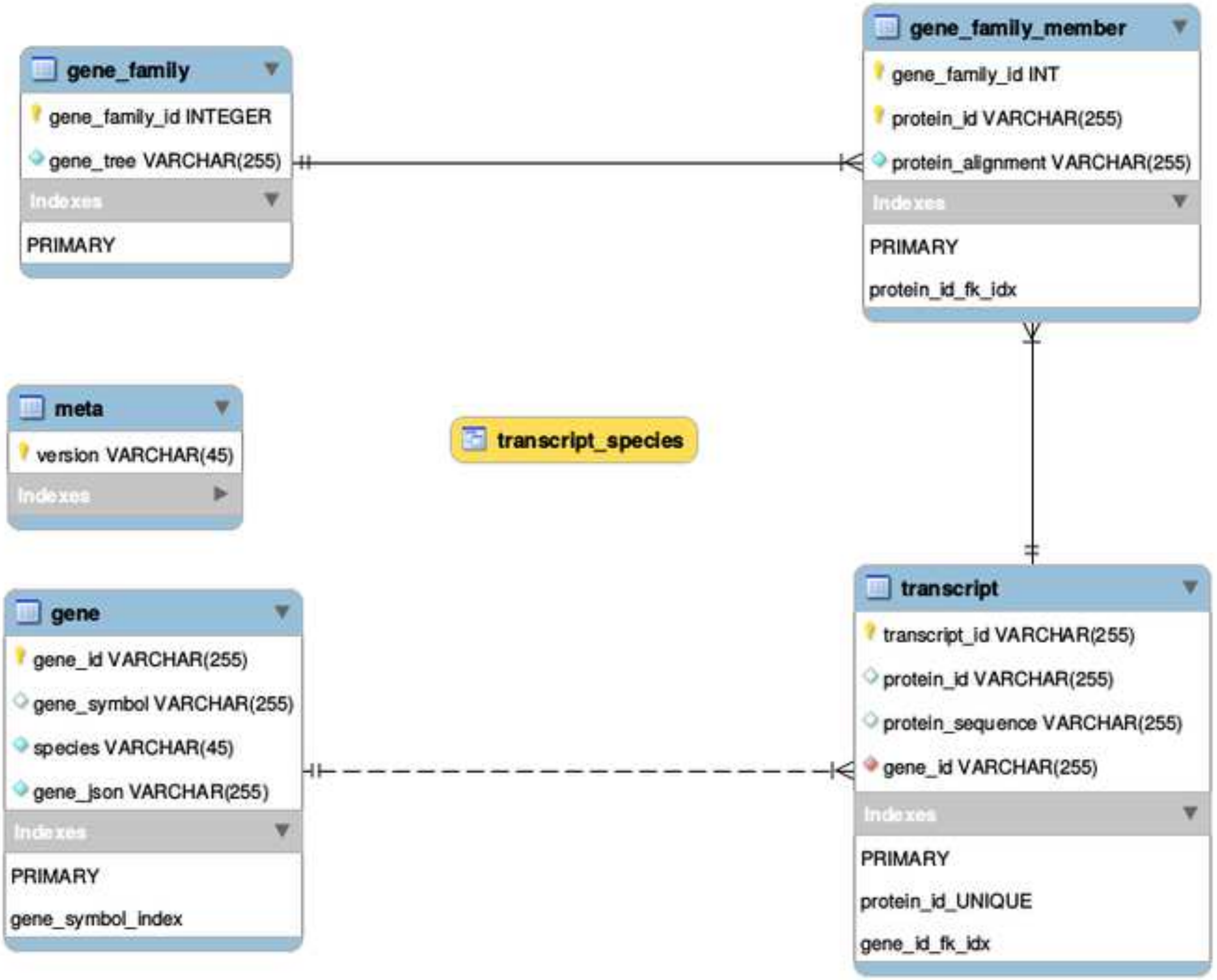

Click here to download Figure 5.png

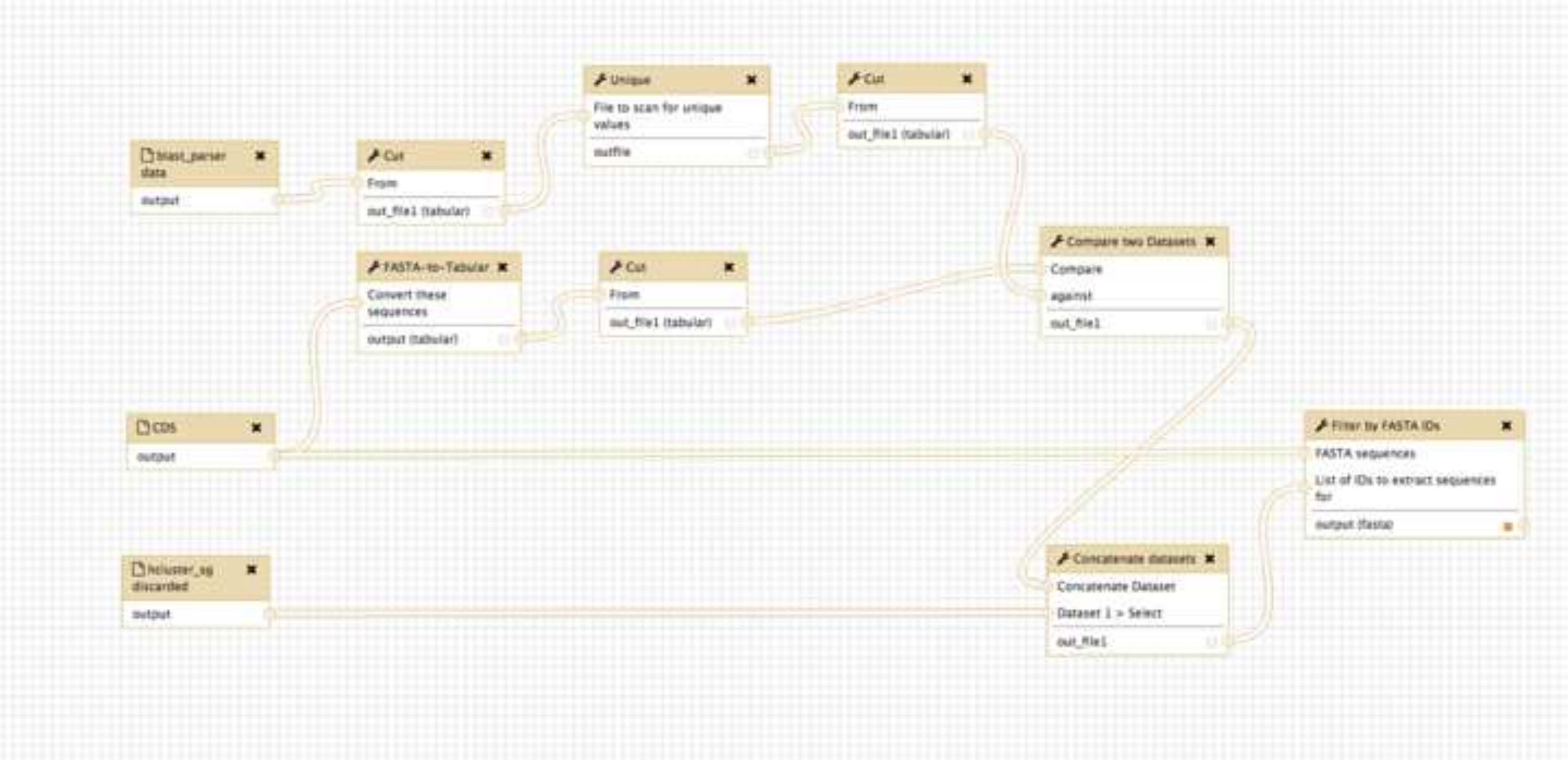

Click here to download Figure 6.png

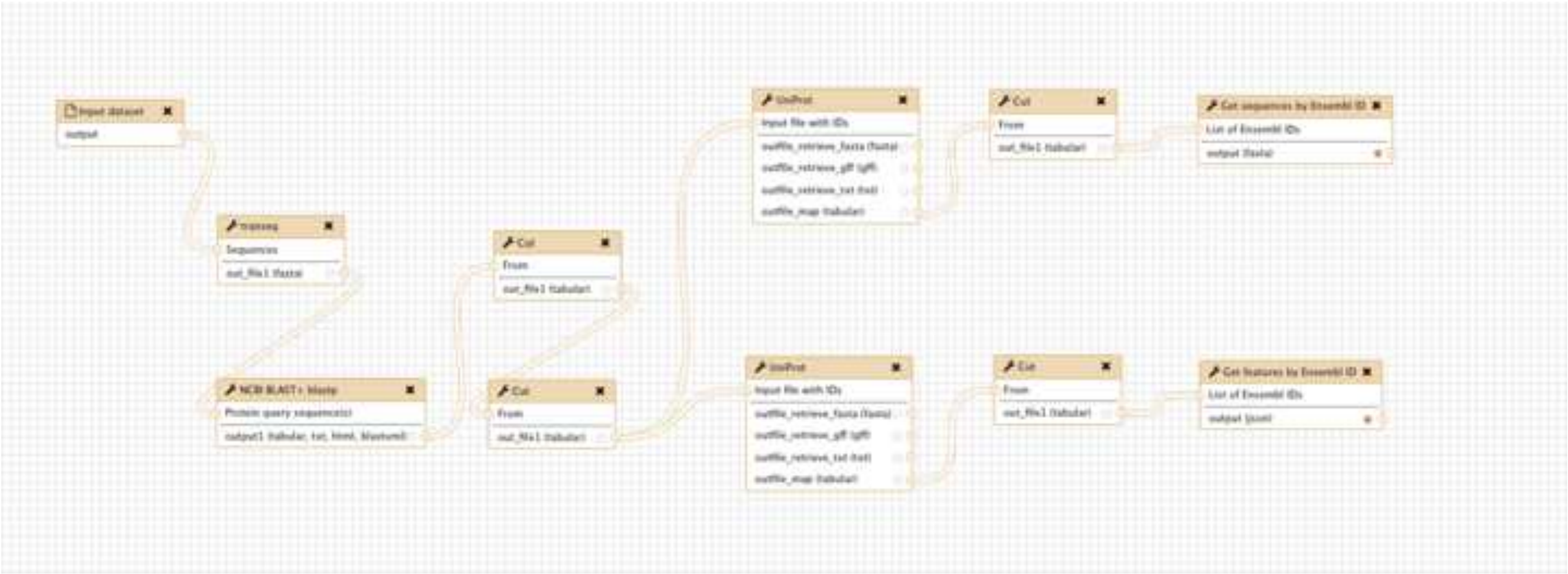

Click here to download Figure 7.png

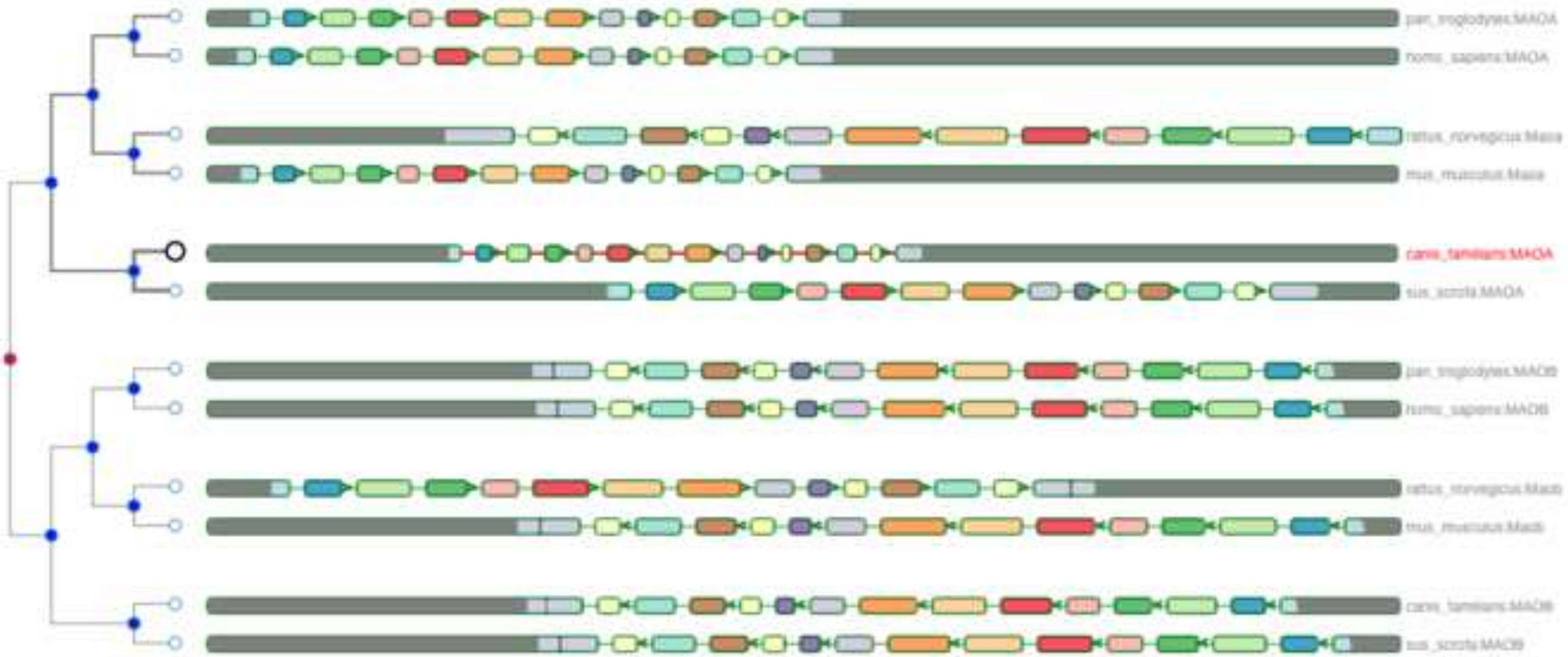

Click here to download Figure 8.png

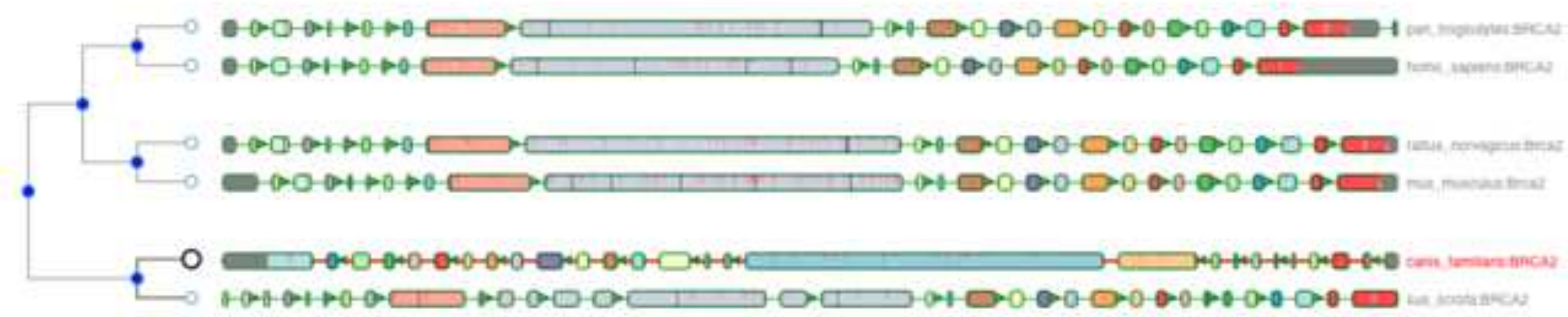

Click here to download Figure 9.png

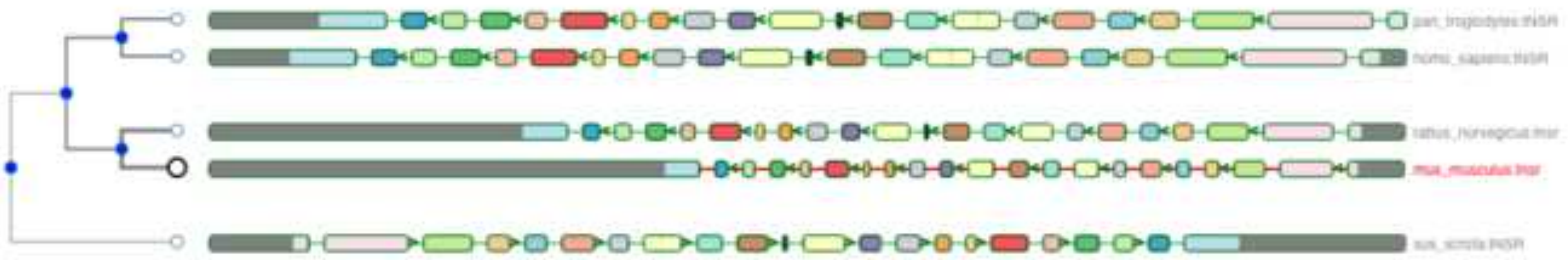

Click here to access/download
Supplementary Material
Additional File 1.docx

 Click here to download Personal Cover cover_letter.doc

Anil S. Thanki

Earlham Institute
Norwich Research Park
Norwich
NR4 7UZ
UK

15th December 2017

Dear Editor,

We wish to submit a new manuscript entitled "**GeneSeqToFamily: a Galaxy workflow to find gene families based on the Ensembl Compara GeneTrees"** for consideration by GigaScience.

We confirm that this work is original and has not been published in any journal nor is it currently under consideration for publication elsewhere.

In this paper, we report on a new Galaxy workflow to find homologous genes and gene trees from a set of genes of interest. The paper should be of interest to readers in the areas of comparative genomics and evolution genomics.

This workflow provides an alternative way to run the Ensembl Compara pipeline in Galaxy without using the command-line, while still providing the flexibility to tailor the analysis by changing configurations and tools if necessary. We would like to publish this manuscript in GigaScience considering its interest and collaboration with the Galaxy community, so it could be part of the **Galaxy Series** of GigaScience.

Please address all correspondence concerning this manuscript to me at Anil.Thanki@earlham.ac.uk or Robert Davey at Robert.Davey@earlham.ac.uk .

Thank you for your consideration of this manuscript.

Sincerely,

Anil S. Thanki
