# Supplementary material for: GeneSeqToFamily: a Galaxy workflow to find gene families based on the Ensembl Compara GeneTrees pipeline
Source: Gigascience. 2018 Feb 7;7(3):giy005. doi: 10.1093/gigascience/giy005 (PMC5863215; doi:10.1093/gigascience/giy005)
Supplement: Supplemental material [file giy005_supp.docx]

# **GeneSeqToFamily: a Galaxy workflow to find gene families based on the Ensembl Compara GeneTrees - Additional File 1**

Anil S. Thanki^1^, Nicola Soranzo^1^, Wilfried Haerty^1^, Robert P. Davey^1^

1. Earlham Institute (EI), Norwich Research Park, Norwich NR4 7UZ, UK

The main aim of the GeneSeqToFamily workflow is to allow the reliable and reproducible identification of gene families across a defined set of species. To assess the robustness of our workflow and compare it with other pipelines, we analysed a large dataset of protein sequences of various species from Quest for Orthologs (QfO) [[1]](https://paperpile.com/c/QmaN2f/dxZl).

We downloaded the sequences for all the 66 species from the QfO Reference Proteomes (version 5; 2011-04), for a total of 754,149 sequences. We then ran the GeneSeqToFamily workflow on each dataset with the set of parameters specified in Table 2, and filtered out matching paralogs from gene families. The obtained results have been submitted back to the QfO benchmarking service (<http://orthology.benchmarkservice.org>) for comparing predictions [[2]](https://paperpile.com/c/QmaN2f/xEwz). The default GeneSeqToFamily workflow predicts gene families including both homologs and paralogs. We removed paralogs from the results as these are not part of the QfO gold standard and would therefore negatively affect the Positive Predictive Value Rate (PPVR).

Figures 1 to 7 show the results of GeneSeqToFamily in QfO benchmarking compared with other tools using the SwissTree method, for various BLASTP and hcluster_sg parameters as shown in Table 2. The figures show that the orthologs predicted from our workflow are directly comparable with prediction of other tools on QfO benchmarking service.

There are caveats, however:

- Since proteomes vary in quality, the results on the benchmark dataset may not entirely reflect the quality of orthology assignments.
- Because of the phylogenetic diversity of the species used in QfO (which includes vertebrates, plants and prokaryotes), it is necessary to provide a so-called category file to run hcluster_sg optimally. Unfortunately neither this file, or the parameters for its compilation, are available from Ensembl Compara, so we cannot perform a like-for-like comparison with it.
- In these examples, True Positive rate is overall better than most of the other tools, but Positive Predictive Value rate is lower, mainly because QfO is designed to assess the specificity and sensitivity of pipelines in calling one 1:1 orthologs, whereas the output of our workflow includes gene families with 1:many and many:many orthologs, which are counted as False Positives by QfO. We cross checked some of these False Positive orthologs with Ensembl Compara database [[3]](https://paperpile.com/c/QmaN2f/JdLm) and found that they are actual orthologs (Table 1).

The decision of whether to favour a skewed or a balanced approach to the precision-recall trade-off strongly depends on the analysis goals, so we leave it up to the user of the workflow to choose the parameters based on the results they want to achieve. Here we provided various examples of parameters we tried for BLASTP and hcluster_sg to compare results.

In conclusion, we are confident that GeneSeqToFamily, based on its comparable true positive rate, and based on the 1:many issue above, is working as expected and of value to the community to assess gene families.

Table 1: Examples of ortholog pairs which are counted as False Positives by QfO benchmarking, but are considered orthologs in the Ensembl Compara database.

| **QfO reference gene tree** | **Gene ID** | **Gene ID** | **Orthology type** | **Link** |
| --- | --- | --- | --- | --- |
| HOX | ENSTRUG00000009562 | ENSXETG00000000724 | 1:many | [[4]](https://paperpile.com/c/QmaN2f/PTOy) |
| HOX | ENSTRUG00000004120 | ENSXETG00000023472 | 1:many | [[5]](https://paperpile.com/c/QmaN2f/vsWa) |
| POP | ENSMODG00000018205 | ENSCING00000000496 | 1:many | [[6]](https://paperpile.com/c/QmaN2f/EJHj) |
| POP | ENSMODG00000018033 | ENSTRUG00000008719 | 1:many | [[7]](https://paperpile.com/c/QmaN2f/eK15) |
|  |  | ENSOANG00000004180 | many:many |  |
|  |  | ENSMMUG00000014471 | many:many |  |
|  |  | ENSPTRG00000018454 | many:many |  |
|  |  | ENSCAFG00000003600 | many:many |  |
|  |  | ENSBTAG00000018790 | many:many |  |
|  |  | ENSXETG00000013301 | many:many |  |
| POP | ENSMODG00000018034 | ENSOANG00000004179 | many:many | [[8]](https://paperpile.com/c/QmaN2f/FDEd) |
|  |  | ENSMMUG00000014473 | many:many |  |
|  |  | ENSPTRG00000018455 | many:many |  |
|  |  | ENSXETG00000024466 | many:many |  |
| POP | ENSTRUG00000015933 | ENSCING00000000496 | 1:many | [[9]](https://paperpile.com/c/QmaN2f/H9Hk) |
| POP | ENSTRUG00000008719 | ENSOANG00000004179 | 1:many | [[10]](https://paperpile.com/c/QmaN2f/UPQe) |
|  |  | ENSMMUG00000014473 | 1:many |  |
|  |  | ENSPTRG00000018455 | 1:many |  |
|  |  | ENSXETG00000024466 | 1:many |  |
| POP | ENSCING00000000496 | ENSMMUG00000000905 | 1:many | [[11]](https://paperpile.com/c/QmaN2f/mqg8) |
|  |  | ENSPTRG00000015259 | 1:many |  |
|  |  | ENSBTAG00000005604 | 1:many |  |
|  |  | ENSXETG00000018064 | 1:many |  |

Table 2: Set of parameters used in BLASTP and hcluster_sg to compare results. BLASTP was configured with maximum number of HSPs set to 1, and hcluster_sg with single link clusters set to ‘no’ and maximum size set to 500.

| **Tool** | **Parameter** | **Parameter set** | | | | | |
| --- | --- | --- | --- | --- | --- | --- | --- |
|  |  | **A** | **B** | **C** | **D** | **E** | **F** |
| BLASTP | expectation value cutoff | 1e-03 | 1e-03 | 1e-03 | 1e-10 | 1e-10 | 1e-10 |
|  | Query coverage per hsp | 0 | 0 | 90 | 0 | 0 | 90 |
| hcluster_sg | Minimum edge weight | 0 | 20 | 0 | 0 | 20 | 20 |
|  | Minimum edge density between a join | 0.34 | 0.50 | 0.34 | 0.34 | 0.50 | 0.50 |


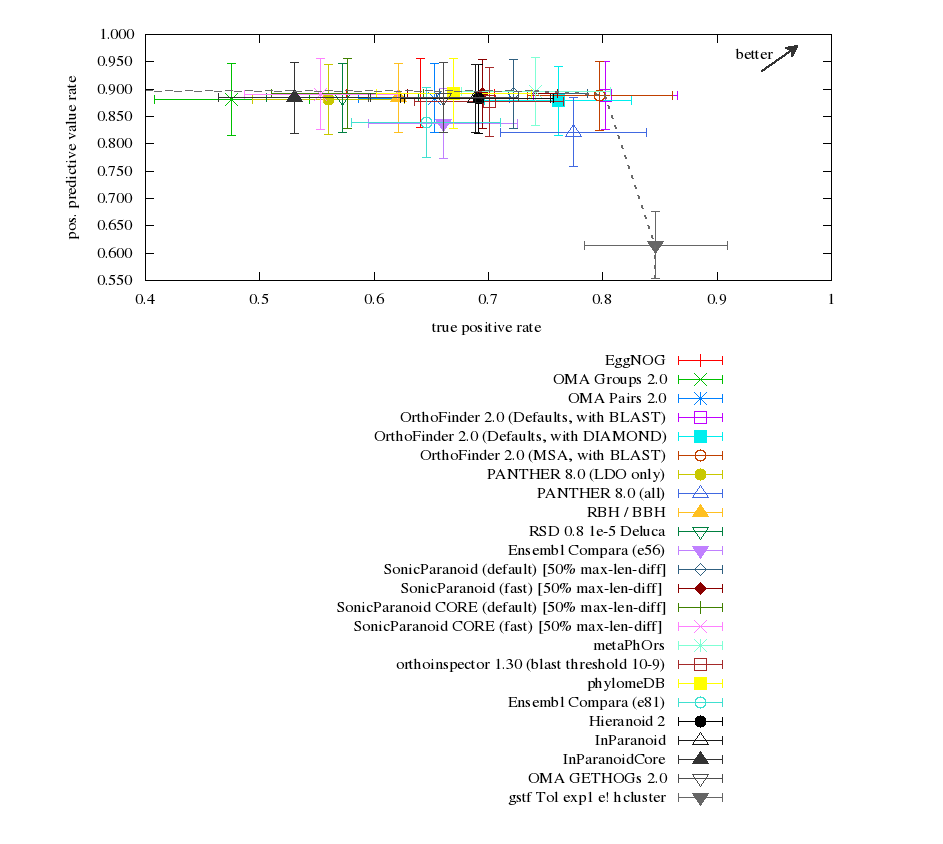


Figure 1: Showing results for benchmarking on Quest for Orthologs’ using parameters shown in Parameter Set A <http://orthology.benchmarkservice.org/cgi-bin/gateway.pl?f=CheckResults&p1=2569682351ea7dfff3d5b083>


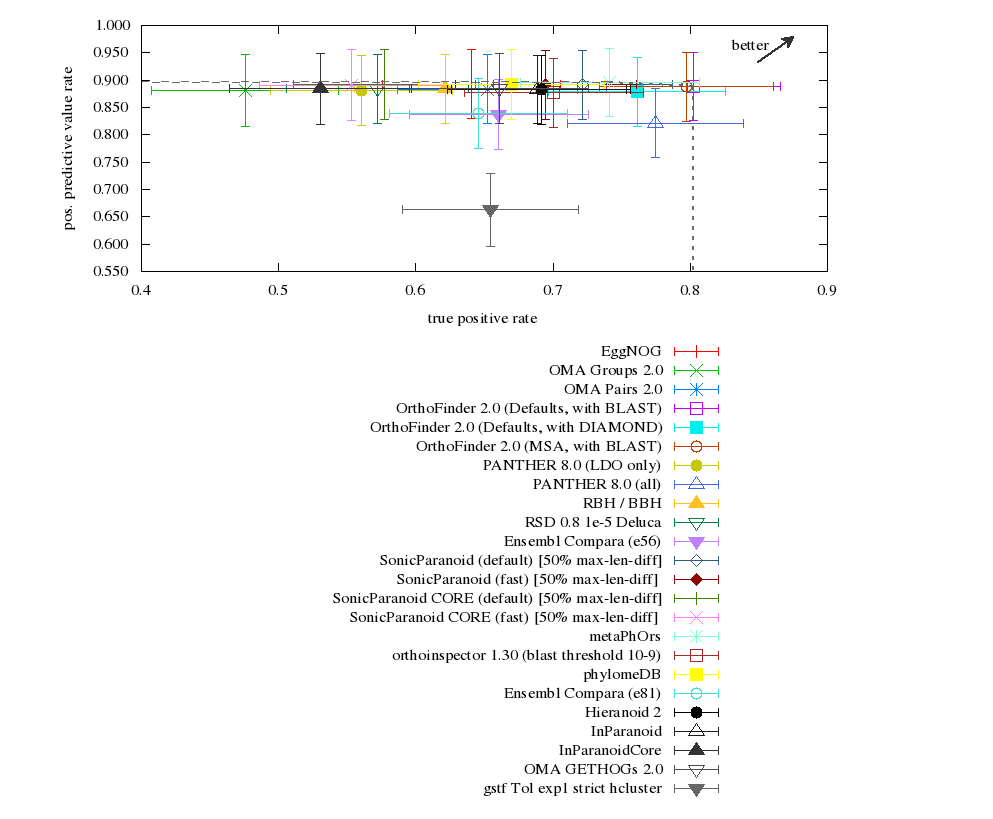


Figure 2: Showing results for benchmarking on Quest for Orthologs’ using parameters shown in Parameter Set B

<http://orthology.benchmarkservice.org/cgi-bin/gateway.pl?f=CheckResults&p1=1038fba4ba15c369b3d25541>


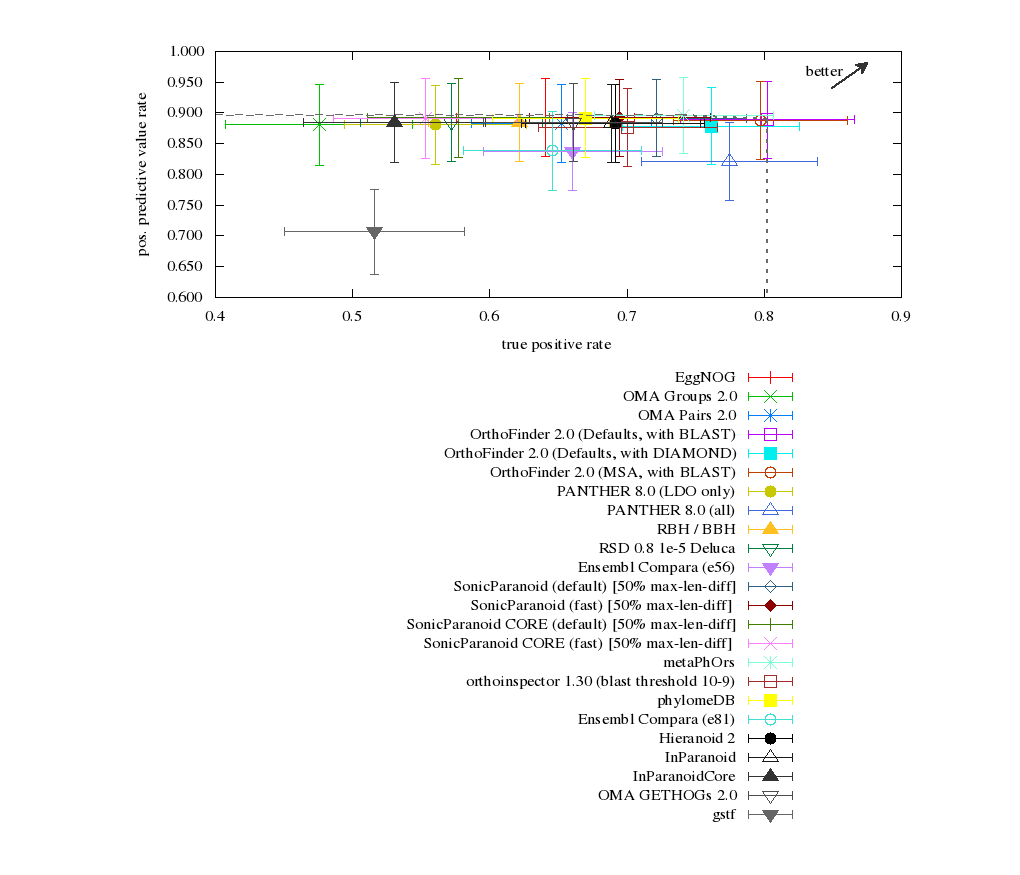


Figure 3: Showing results for benchmarking on Quest for Orthologs’ using parameters shown in Parameter Set C <http://orthology.benchmarkservice.org/cgi-bin/gateway.pl?f=CheckResults&p1=ec4d223d24e0a7f54edd3692>


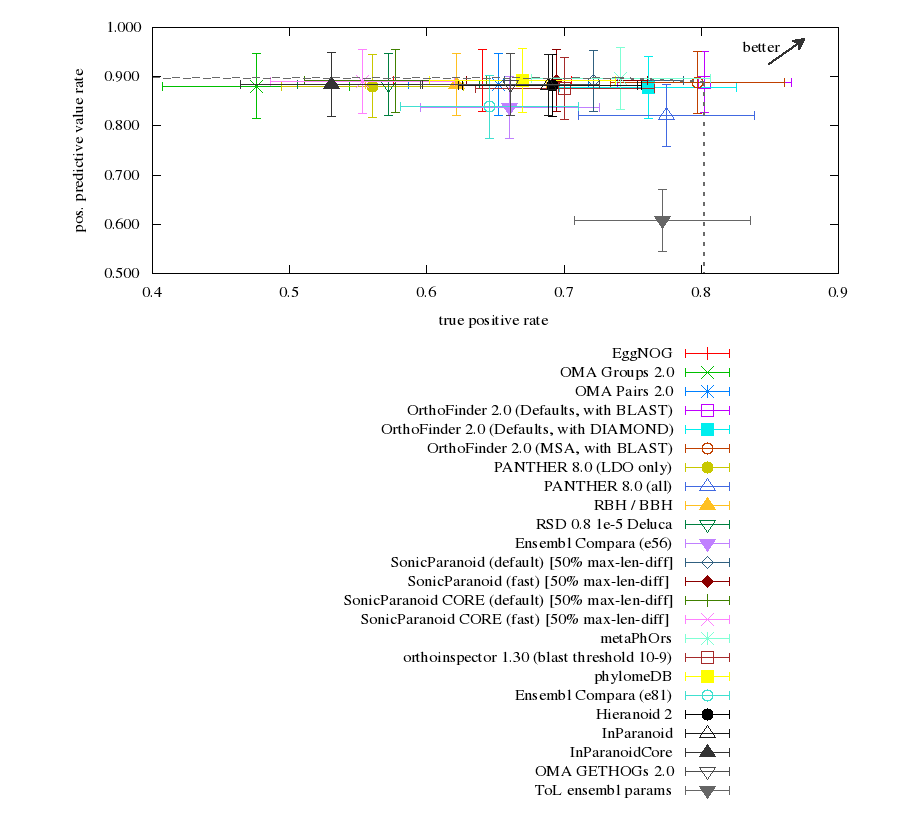


Figure 4: Showing results for benchmarking on Quest for Orthologs’ using parameters shown in Parameter Set D

<http://orthology.benchmarkservice.org/cgi-bin/gateway.pl?f=CheckResults&p1=dc81a95f182f5b5bee2dab3f>


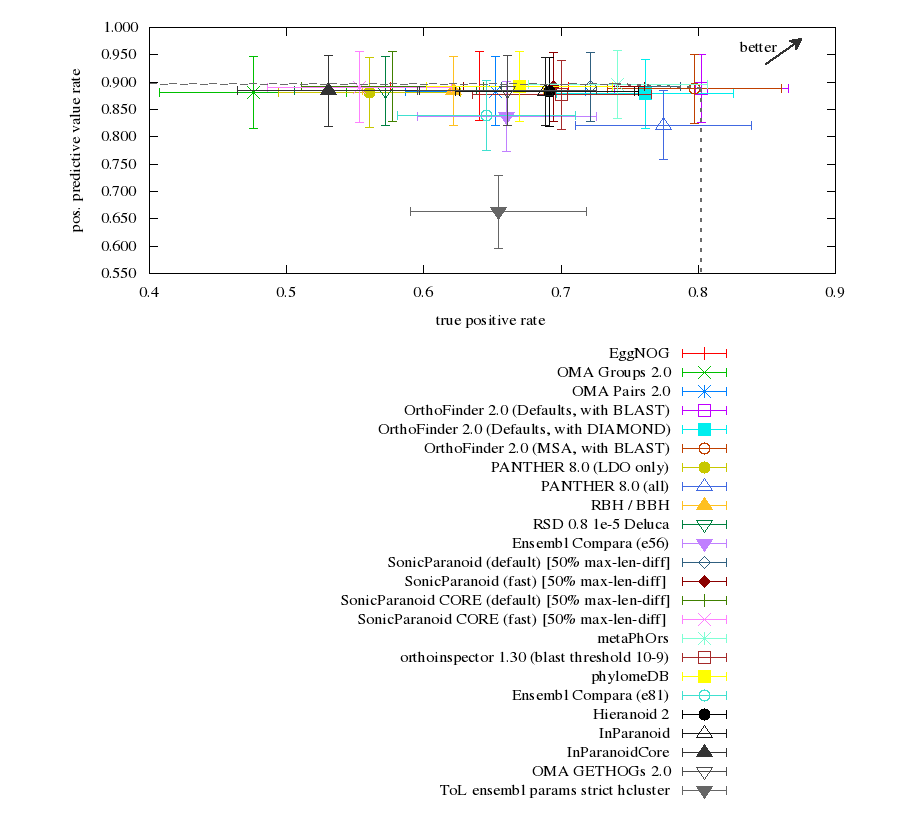


Figure 5: Showing results for benchmarking on Quest for Orthologs’ using parameters shown in Parameter Set E <http://orthology.benchmarkservice.org/cgi-bin/gateway.pl?f=CheckResults&p1=9d35f843bcae077e917a6452>


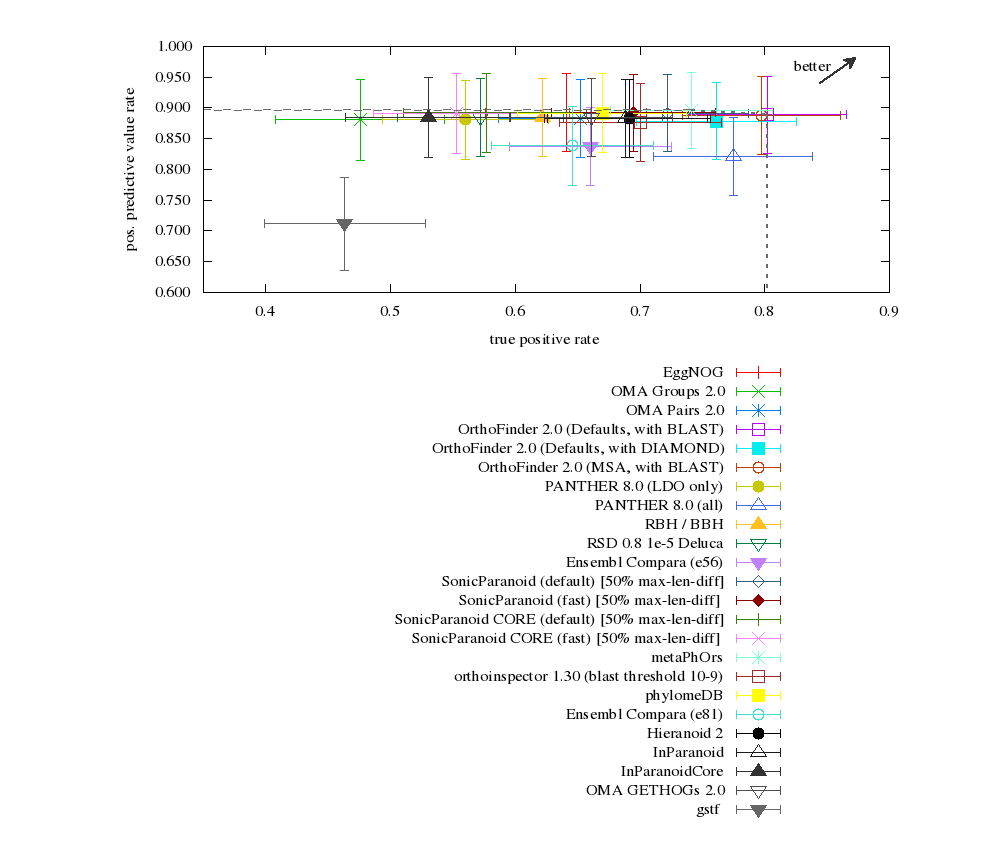


Figure 6: Showing results for benchmarking on Quest for Orthologs’ using parameters shown in Parameter Set F http://orthology.benchmarkservice.org/cgi-bin/gateway.pl?f=CheckResults&p1=0cbd5a0267b87491252348d6

References:

[1. Kuzniar A, van Ham RCHJ, Pongor S, Leunissen JAM. The quest for orthologs: finding the corresponding gene across genomes. Trends Genet. 2008;24:539–51.](http://paperpile.com/b/QmaN2f/dxZl)

[2. Altenhoff AM, Boeckmann B, Capella-Gutierrez S, Dalquen DA, DeLuca T, Forslund K, et al. Standardized benchmarking in the quest for orthologs. Nat. Methods. 2016;13:425–30.](http://paperpile.com/b/QmaN2f/xEwz)

[3. Herrero J, Muffato M, Beal K, Fitzgerald S, Gordon L, Pignatelli M, et al. Ensembl comparative genomics resources. Database [Internet]. Oxford University Press; 2016 [cited 2017 Dec 5];2016. Available from:](http://paperpile.com/b/QmaN2f/JdLm) <https://academic.oup.com/database/article-pdf/doi/10.1093/database/bav096/8222278/bav096.pdf>

[4. Gene: hoxa9b (ENSTRUG00000009562) - Orthologues - Takifugu rubripes - Ensembl genome browser 90 [Internet]. [cited 2017 Dec 4]. Available from:](http://paperpile.com/b/QmaN2f/PTOy) <http://www.ensembl.org/Takifugu_rubripes/Gene/Compara_Ortholog?db=core;g=ENSTRUG00000009562;r=scaffold_39:583315-598694>

[5. Website [Internet]. [cited 2017 Dec 4]. Available from:](http://paperpile.com/b/QmaN2f/vsWa) <http://www.ensembl.org/Takifugu_rubripes/Gene/Compara_Ortholog?db=core;g=ENSTRUG00000004120;r=scaffold_66:162811-173892>

[6. Gene: POPDC2 (ENSMODG00000018205) - Orthologues - Monodelphis domestica - Ensembl genome browser 90 [Internet]. [cited 2017 Dec 4]. Available from:](http://paperpile.com/b/QmaN2f/EJHj) <http://www.ensembl.org/Monodelphis_domestica/Gene/Compara_Ortholog?db=core;g=ENSMODG00000018205;r=4:83393959-83418098;t=ENSMODT00000023099>

[7. Gene: ENSMODG00000018033 - Orthologues - Monodelphis domestica - Ensembl genome browser 90 [Internet]. [cited 2017 Dec 4]. Available from:](http://paperpile.com/b/QmaN2f/eK15) <http://www.ensembl.org/Monodelphis_domestica/Gene/Compara_Ortholog?db=core;g=ENSMODG00000018033;r=2:369226598-369265423;t=ENSMODT00000022877>

[8. Website [Internet]. [cited 2017 Dec 4]. Available from:](http://paperpile.com/b/QmaN2f/FDEd) <http://www.ensembl.org/Monodelphis_domestica/Gene/Compara_Ortholog?db=core;g=ENSMODG00000018034;r=2:369135575-369203972;t=ENSMODT00000022878>

[9. Gene: popdc2 (ENSTRUG00000015933) - Orthologues - Takifugu rubripes - Ensembl genome browser 90 [Internet]. [cited 2017 Dec 4]. Available from:](http://paperpile.com/b/QmaN2f/H9Hk) <http://www.ensembl.org/Takifugu_rubripes/Gene/Compara_Ortholog?db=core;g=ENSTRUG00000015933;r=scaffold_38:1588237-1591665>

[10. Gene: ENSTRUG00000008719 - Orthologues - Takifugu rubripes - Ensembl genome browser 90 [Internet]. [cited 2017 Dec 4]. Available from:](http://paperpile.com/b/QmaN2f/UPQe) <http://www.ensembl.org/Takifugu_rubripes/Gene/Compara_Ortholog?db=core;g=ENSTRUG00000008719;r=scaffold_148:430012-440384>

[11. Gene: ENSCING00000000496 - Orthologues - Ciona intestinalis - Ensembl genome browser 90 [Internet]. [cited 2017 Dec 4]. Available from:](http://paperpile.com/b/QmaN2f/mqg8) <http://www.ensembl.org/Ciona_intestinalis/Gene/Compara_Ortholog?db=core;g=ENSCING00000000496;r=6:2300533-2303507;t=ENSCINT00000000907>
