# Supplementary material for: GeneSeqToFamily: a Galaxy workflow to find gene families based on the Ensembl Compara GeneTrees pipeline
Source: Gigascience. 2018 Feb 7;7(3):giy005. doi: 10.1093/gigascience/giy005 (PMC5863215; doi:10.1093/gigascience/giy005)
Supplement: Response_to_Reviewer_Comments_Original_Submission.docx [file giy005_response_to_reviewer_comments_original_submission.docx]

**Author's Response To Reviewer Comments**

Top of Form

Bottom of Form

Dear Reviewers:

I am pleased to resubmit for publication the revised version of “GeneSeqToFamily: the Ensembl Compara GeneTrees pipeline as a Galaxy workflow.” I appreciated the constructive criticisms of the reviewers. I have addressed each of their concerns as outlined below.

Reviewer #1:

The authors have put together a Galaxy workflow incorporating various tools to be able to perform Ensembl Compara's pipeline for gene family identification and synteny visualization. This in itself is valuable and useful and I have no concerns with the pipeline itself, though I have not tried it.

However, I am unsatisfied with the parameter evaluation and benchmarking:

1 - First, to test the tool, the objective must be clearer defined. Is the goal to find homologs most generally? Full-length homologs? Orthologs? And if orthologs, then orthologs relative to the set of species used as input or something else? This must be explicitly stated for the evaluation and parameter search to be well-defined. The manuscript should also make clear that this goal is the one that utility for reaching is implied, instead of any other goal.

Response:
The main aim of the test is to demonstrate that we are able to identify orthogroups (groups of genes sharing ancestry by descent) across a set of species. In the revised version of the manuscript, we clarified the goals of the tool and its purpose.

2 - The data set used for parameter searches is tiny, just three species, two of which are obscure (how good are assemblies?). This should be done on a much larger set, perhaps use the Quest for Orthologs' reference dataset?

Response:
Although we only used three species, the data set includes over 63,000 genes. The reviewer raises a valid question regarding the quality of the assemblies, i.e. while the mouse genome can be considered of very high quality, both the platypus and Tasmanian devil genome assemblies are of much lower quality. Therefore, we have now run our workflow on two sets of three species, one including high quality assemblies (human, mouse and dog), the second set composed of draft genome assemblies only (pig, platypus and horse). Furthermore, we ran our analysis on all six species to assess the impact of mixed assemblies qualities on the identification of gene families.

3 - Testing six settings does not show that one of the settings consistently give any particular results. The way to do that would be to test each parameter set many times, using different species combinations each time, and check if indeed Analysis 5 parameters consistently yield desired results under a well-defined quality metric (dependent on the goal posts set above).

Response:
As per reviewer’s request, we tested six sets of parameters on the high quality, low quality, and both high and low quality datasets mentioned above. All datasets tested confirm that Analysis 5 returns the optimal results for these datasets. We acknowledge that different datasets might require further tuning, but we believe these parameters to be suitably comprehensive for most analyses.

4 - Testing performance on 23 genes is vastly insufficient. Please look into much larger benchmark sets (e.g. OrthoBench or quest for orthologs' benchmark tool), choose some larger, more diverse (functionally and phylogenetically) set to use as a gold standard, define a metric for success, then show how well the tool works under repeated applications of method settings on different subsets of the data.

Response:
We tried running our workflow with ‘Quest for orthologs’, but the recommended dataset available for this tool is very old (2011). We contacted the author of Quest for Orthologs for the latest release (2017), but there is no gold standard resultant data available to compare to, so maybe we can look into benchmarking this workflow in the future when results from other tools are available to compare with.

5 To be clear, I think this is valuable and worthy of publication, but for any claim of functionality, it must be robustly tested under clear definitions on large-scale diverse and representative data as stated above (which should also have some clear measure of accuracy relative to the gold standard).

Response:
We thank the reviewer for their comments, and we hope that the revised manuscript and associated broader analyses will help readers decide whether this workflow is suitable for their datasets. We feel that the mechanisms that the reviewer kindly suggested for assessing our workflow against a gold standard are inadequate to give a representative assessment. We will continue to monitor the available tools and standards in order to provide support for the usefulness of the workflow.

6 Similarly, please add time and memory consumption statistics for different use cases.

Response:
Time and memory consumption for the workflow depends on the infrastructure it is running on. We ran the Galaxy jobs on an High Performance Computing cluster, where measuring the amount of memory used is tricky. Also, the total time used to complete the pipeline may heavily vary depending on the time spent in the cluster scheduler waiting for resources to be available.

Anyhow, most of the time is spent on the NCBI blastp step, which for the examples in the paper varied between 16 and 24 hours for 6 species with 125,342 sequences.


Reviewer #2:
The article presents GeneSeqToFamily, the Ensembl Compara GeneTrees pipeline as a Galaxy workflow. Authors introduce the importance of studying homologous genes to understand evolution of gene families. They present the Ensembl Compara GeneTrees pipeline and the resulting conversion into a Galaxy workflow named GeneSeqToFamily. This workflow is thus the first "ready-to-use" solution providing information about structural changes within genes using Galaxy. The article is well written and I would like to see it published, I have provided very few notes, comments, and suggestions below.
I don't succeed to find typo or other little errors in the manuscript. Moreover, I don't have any major recommendation. Only few minors remarks, maybe ideas who can be of interest… or not ;)

Article

I particularly like the manner authors have presented workflow tools in the table 1. This is a good manner to summarized components, details and origins of each tool. Moreover, this translate the wish of transparency by authors and the fact they don't want to reinvent the wheel using, if relevant, existing tools.

Response:
We thank the reviewer for their kind comments and agree that reinventing the wheel is something we tried to avoid!

1) It appears to me that selecting the longest CDS per gene is a good idea and I'm wondering if this step cannot be made through the use of "classical" existing Galaxy tools as "Text manipulation" ones.

Response:
It is possible to use other tools, but users would probably have to chain together multiple tools to achieve the same result. By having our own tool we can make sure it works perfectly with different data formats, which could be difficult with other tools.

Concerning clustering steps, I want to propose some ideas, but not sure these are appropriate….

2) Not sure this is relevant but concerning clustering of similar sequences, I'm wondering if the use of algo like Swarm (https://github.com/torognes/swarm) (dedicated to metagenomics amplicon-based ngs data clustering) can be of interest…

Response:
As a first release of the workflow, we mainly concentrated on reproducing the Ensembl Compara pipeline into an easy-to-use Galaxy workflow. But we are grateful to the reviewer for the suggestion of testing alternatives for the stock tools in the workflow, and that is something we intend to look into in the future.

3) Concerning the overall clustering approach and related filtered steps, maybe it can be applied
a) a combination of parallel clustering methods (K-means/PAM/CAH/…) to compare "raw" clustering results and thus eliminate / reduce potential noise (ie sequences moving from one cluster to another when we change the clustering method, translating that this sequence is hard to assign)
b) a clustering affecting bootstrap confidence score to nodes thus giving ideas about relevance of each cluster.

Response:
We are grateful to reviewer for the suggestions for the clustering tools. As mentioned above, in first release of the workflow our aim is to reproduce the Ensembl Compara pipeline into a Galaxy workflow, which we managed to achieve. Improving the workflow by modifying the clustering approach is something we can look into in a future release of the workflow.

4) you propose the use of the Newick format. Maybe, as for the Philoviz visualization plugin, it would be interesting to add Nexus and phyloxml datatypes?

Response:
This workflow requires Newick format for the species tree because TreeBeST accepts input only in this format. There are several tools available to convert from Nexus and PhyloXML to Newick format, e.g. http://biopython.org/wiki/Phylo , so users who are producing data in these formats can perform the conversion before uploading them to Galaxy.

6) Maybe authors can give more details more aspects related to particular issues related to
a) alternative transcripts as it appears to me that ""just"" changing the max_target_seqs value from 3 to 4 seems to be quite "light" and

Response:
We agree with the reviewer that the explanation for changing the max_target_seqs value was a bit unclear in the manuscript, we have rewritten this part and reviewers will hopefully find it more satisfying.

b) working on non-model organisms.

Response:
With the significant reduction of sequencing costs, the proportion of fully sequenced genomes from non-model organisms has been steadily increasing. The analysis of the evolution of gene families across many species, including non-model organisms, allows us to assess the quality of the genome assemblies and associated annotations. We can also derive observations linking the expansion and contraction of gene families, as well as detect signals of positive selection in genes with 1:1 orthologs to specific physiological and behavioural adaptations (e.g. association between beta-keratin and shell evolution in turtles; Li et al. 2013. Genome Biol Evol. 5(5):923-33); taste receptors in whales - Feng et al. 2014 Genome Biol Evol. 6(6):1254-65). We have added such details into the manuscript.

Unfortunately, I don't have taking time to implement the GeneSeqToFamily tools and workflow to a dedicated Galaxy instance. I would have appreciated to have a link giving access to a testing GeneSeqToFamily Galaxy instance. Maybe creating and pointing a dedicated Docker based Galaxy flavor can be of interest, thus giving an easy way to deploy a GeneSeqToFamily local Galaxy instance.

Response:
We have created a virtual image for testing the workflow, available [1], which is ready to be used with VirtualBox, and is supplied with necessary tools and plugins installed for the workflow. We will look into producing a Docker image for the same purpose in future.

8) Moreover, regarding the https://github.com/TGAC/earlham-galaxytools/tree/master/workflows/GeneSeqToFamily github link, the readme file "only" explain GeneSeqToFamily workflow without instructions on how to deploy it.

Response:
We have updated the readme.md file for the workflow with the list of tools required and instructions explaining how to install the workflow.

9) Regarding tools on the toolshed, it would be nice to have a repository called "suite_GeneSeqToFamily" or something like that to easily install all GeneSeqToFamily related Galaxy tools without installing it one by one like for the "suite_stacks" tools for example.

Response:
In the forthcoming Galaxy release 17.09 it will be possible to install a workflow together with the needed tools, so we hope that this feature is going to solve this issue without having to create a “suite” repository.


[1] Galaxy with GeneSeqToFamily http://repos.tgac.ac.uk/vms/Galaxy_with_GeneSeqToFamily.ova
