# Supplementary material for: GeneSeqToFamily: a Galaxy workflow to find gene families based on the Ensembl Compara GeneTrees pipeline
Source: Gigascience. 2018 Feb 7;7(3):giy005. doi: 10.1093/gigascience/giy005 (PMC5863215; doi:10.1093/gigascience/giy005)
Supplement: Response_to_Reviewer_Comments_Revision_1.docx [file giy005_response_to_reviewer_comments_revision_1.docx]

**Author's Response To Reviewer Comments**

Top of Form

Bottom of Form

>>>Original comment:
>>>First, to test the tool, the objective must be clearer defined. Is the goal to find
homologs most generally? Full-length homologs? Orthologs? And if orthologs, then
orthologs relative to the set of species used as input or something else? This must be
explicitly stated for the evaluation and parameter search to be well-defined. The
manuscript should also make clear that this goal is the one that utility for reaching is
implied, instead of any other goal.

>>Author response:
>>The main aim of the test is to demonstrate that we are able to identify orthogroups
(groups of genes sharing ancestry by descent) across a set of species. In the revised
version of the manuscript, we clarified the goals of the tool and its purpose.

>Reviewer response:
>This is still unclear. What paper defined the term "orthogroup" and where do you cite it for this purpose? Where in this MS is that term used? The introduction still does not define gene family, and does not say if these are just homologous or whether they will be orthologous relative to a particular ancestral taxon, and if so which one. The introduction needs a clear definition of gene family, and the method needs to be stated as providing gene families according to this particular definition. Otherwise any data on performance are useless, because it is not possible to say whether or not the stated goals have been reached. Please revise and add this.

Author Response:
We used the same definition of orthogroups as Gabaldón and Koonin (Nature Rev. Genet. 2013), and we have added the citation in the revised version of the manuscript. In addition, we have clarified the introduction by adding the definitions of “gene family” and “homologous genes”. This workflow focuses on the discovery and characterisation of gene families, not specifically on the benchmarking of orthologs or paralogs.


>>>Original comment:
>>>The data set used for parameter searches is tiny, just three species, two of which
are obscure (how good are assemblies?). This should be done on a much larger set,
perhaps use the Quest for Orthologs' reference dataset?

>>Author response:
>>Although we only used three species, the data set includes over 63,000 genes. The
reviewer raises a valid question regarding the quality of the assemblies, i.e. while the
mouse genome can be considered of very high quality, both the platypus and
Tasmanian devil genome assemblies are of much lower quality. Therefore, we have
now run our workflow on two sets of three species, one including high quality
assemblies (human, mouse and dog), the second set composed of draft genome
assemblies only (pig, platypus and horse). Furthermore, we ran our analysis on all six
species to assess the impact of mixed assemblies qualities on the identification of
gene families.

>Reviewer response:
>One set of three species, another set of three species, one set of six species - who does this sort of tiny analysis in real practice? Furthermore, just by chance with this few you might perceive trends that would disappear with a more balanced benchmark. This is not sufficiently addressing my concern. Please revise to do so.

Author Response:
Here we chose 3 species with high quality and 3 species with low quality of genome assembly to check the impact of quality of a genome on how the workflow is able to discern gene families. Additionally, with the increasing number of genomes released, many being early assemblies, this analysis is of direct interest to the intended users of our workflow, highlighting the impact of assembly quality on gene family detection. Following the reviewer's comment we also performed an analysis on the 66 species from Quest for Orthologs’ (QfO) reference proteome (see below).

>>>Original comment:
>>>Testing six settings does not show that one of the settings consistently give any
particular results. The way to do that would be to test each parameter set many times,
using different species combinations each time, and check if indeed Analysis 5
parameters consistently yield desired results under a well-defined quality metric
(dependent on the goal posts set above).

>>Author response:
>>As per reviewer's request, we tested six sets of parameters on the high quality, low
quality, and both high and low quality datasets mentioned above. All datasets tested
confirm that Analysis 5 returns the optimal results for these datasets. We acknowledge
that different datasets might require further tuning, but we believe these parameters to
be suitably comprehensive for most analyses.

>Reviewer response:
>So since my concern 2) was not yet addressed, 3) also was not, since that would require being able to sample the species space more than just 3+3 species allow. I don't understand why what I asked for was not done here? Since this should be an easily usable pipeline for wide audience, doing so should not be so difficult. Please revise.

Author Response:
As per reviewer’s request we ran our workflow on QfO reference proteome using various sets of parameters. Due to the size of the results, we provide an additional file alongside our manuscript informing users on the impact of parameter choices. However, we would like to reiterate that the GeneSeqToFamily workflow is designed to identify gene families, including both 1:1 orthologs and paralogs. During our analysis, we identified two factors that adversely affect Positive Prediction Value Rate achieved using the GeneSeqToFamily pipeline.
Firstly, QfO is designed to assess the specificity and sensitivity of pipelines in calling 1:1 orthologs, whereas the output of our workflow includes gene families with 1:many and many:many orthologs. These 1:many orthologs are counted towards the False Positive (FP) rate by QfO. To validate our findings, we manually checked these FPs against the orthologs listed in the Ensembl Compara database at EBI. We did indeed find that our FPs are listed as 1:many or many:many orthologs in Compara, and therefore we conclude that because these are not recorded as such in the QfO output, there is some data cleaning (most likely tree reconciliation) that is carried out prior to submitting to QfO.
Secondly, because of the phylogenetic diversity of the species used in QfO, it is necessary to provide a category file to run hcluster_sg optimally. Unfortunately neither this file, or the parameters for its compilation are available from Ensembl Compara, so we cannot perform a like-for-like comparison. We are confident that GeneSeqToFamily, based on its comparable true positive rate, and based on the 1:many issue above, is working as expected and of value to the community to assess gene families.

>>>Original comment:
>>>Testing performance on 23 genes is vastly insufficient. Please look into much larger
benchmark sets (e.g. OrthoBench or quest for orthologs' benchmark tool), choose
some larger, more diverse (functionally and phylogenetically) set to use as a gold
standard, define a metric for success, then show how well the tool works under
repeated applications of method settings on different subsets of the data.

>>Author response:
>>We tried running our workflow with 'Quest for orthologs', but the recommended dataset
available for this tool is very old (2011). We contacted the author of Quest for
Orthologs for the latest release (2017), but there is no gold standard resultant data
available to compare to, so maybe we can look into benchmarking this workflow in the
future when results from other tools are available to compare with.

>Reviewer response:
>This makes no sense. It may be old (2011 is old?) but it is still 60+ chosen species, so much more than you currently use. Furthermore it was specifically designed (with input from specialists) to be such a gold standard, and if it has not changed much, this is because discussions on whether to add/subtract species since have not lead to any consensus that doing so is called for. It is eminently usable, have been so used in other contexts, and works fine for this purpose. If you prefer another set of similar size you can of course use that, but you need to benchmark this on a non-toy dataset. Please revise.

Author Response:
This request should be satisfied by our previous answers.

>>>Original comment:
>>>To be clear, I think this is valuable and worthy of publication, but for any claim of
functionality, it must be robustly tested under clear definitions on large-scale diverse
and representative data as stated above (which should also have some clear measure
of accuracy relative to the gold standard).

>>Author response:
>>We thank the reviewer for their comments, and we hope that the revised manuscript
and associated broader analyses will help readers decide whether this workflow is
suitable for their datasets. We feel that the mechanisms that the reviewer kindly
suggested for assessing our workflow against a gold standard are inadequate to give a
representative assessment. We will continue to monitor the available tools and
standards in order to provide support for the usefulness of the workflow.

>Reviewer response:
>This is just referencing 5 above. As with that, you need to redo on a properly large benchmark.

Author Response:
As mentioned above, this request should also be satisfied by our previous answers. However, we feel we should comment that the results of QfO are hugely determinant on finding the exact parameters that give the best results for this singular benchmark. Whilst QfO is clearly valuable to inform confidence on already known orthologs, we feel GeneSeqToFamily comprises the best of both worlds - high accuracy (1:many FP issue aside, reported above) and the ability to characterise unknown gene families.

>>>Original comment:
>>> Similarly, please add time and memory consumption statistics for different use cases.

>>Author response:
>>Time and memory consumption for the workflow depends on the infrastructure it is
running on. We ran the Galaxy jobs on an High Performance Computing cluster, where
measuring the amount of memory used is tricky. Also, the total time used to complete
the pipeline may heavily vary depending on the time spent in the cluster scheduler
waiting for resources to be available.
Anyhow, most of the time is spent on the NCBI blastp step, which for the examples in
the paper varied between 16 and 24 hours for 6 species with 125,342 sequences.

>Reviewer response:
>The cluster should have a queuing system for its nodes, which if anything ought to make assessing memory use easier. 16-24 hours using how many nodes/cores? Please add these numbers to the revised manuscript.

Author Response:
We’ve now collected runtime and memory statistics for a typical run of the GeneSeqToFamily workflow (up to the hcluster_sg step, where gene families are determined) on the QfO proteomes.
Over 99% of the runtime was spent on BLASTP, which was run in parallel on the 66 protein FASTA files (one for each reference species). The average runtime of BLASTP, using 4 cores, was 735 minutes, with a maximum of 4303 min.
The step using the majority of the pipeline memory allocation was hcluster_sg with 2.32 GB.
