# Supplementary material for: GeneSeqToFamily: a Galaxy workflow to find gene families based on the Ensembl Compara GeneTrees pipeline
Source: Gigascience. 2018 Feb 7;7(3):giy005. doi: 10.1093/gigascience/giy005 (PMC5863215; doi:10.1093/gigascience/giy005)
Supplement: Reviewer_1_Report_(Original_Submission) -- Kristoffer Forslund — 24 Apr 2017 Reviewed [file giy005_reviewer_1_report_(original_submission).docx]

**Reviewer Report**

**Title:** GeneSeqToFamily: a Galaxy workflow to find gene families based on the Ensembl Compara GeneTrees

**Version:** Original Submission	**Date:** 24 Apr 2017

**Reviewer name:** Kristoffer Forslund

**Reviewer Comments to Author:**

he authors have put together a Galaxy workflow incorporating various tools to be able to perform Ensembl Compara's pipeline for gene family identification and synteny visualization. This in itself is valuable and useful and I have no concerns with the pipeline itself, though I have not tried it.

However, I am unsatisfied with the parameter evaluation and benchmarking:

- First, to test the tool, the objective must be clearer defined. Is the goal to find homologs most generally? Full-length homologs? Orthologs? And if orthologs, then orthologs relative to the set of species used as input or something else? This must be explicitly stated for the evaluation and parameter search to be well-defined. The manuscript should also make clear that this goal is the one that utility for reaching is implied, instead of any other goal.

- The data set used for parameter searches is tiny, just three species, two of which are obscure (how good are assemblies?). This should be done on a much larger set, perhaps use the Quest for Orthologs' reference dataset?

- Testing six settings does not show that one of the settings consistently give any particular results. The way to do that would be to test each parameter set many times, using different species combinations each time, and check if indeed Analysis 5 parameters consistently yield desired results under a well-defined quality metric (dependent on the goal posts set above).

- Testing performance on 23 genes is vastly insufficient. Please look into much larger benchmark sets (e.g. OrthoBench or quest for orthologs' benchmark tool), choose some larger, more diverse (functionally and phylogenetically) set to use as a gold standard, define a metric for success, then show how well the tool works under repeated applications of method settings on different subsets of the data.

To be clear, I think this is valuable and worthy of publication, but for any claim of functionality, it must be robustly tested under clear definitions on large-scale diverse and representative data as stated above (which should also have some clear measure of accuracy relative to the gold standard).

Similarly, please add time and memory consumption statistics for different use cases.

**Level of Interest**

Please indicate how interesting you found the manuscript: An article whose findings are important to those with closely related research interests

**Quality of Written English**

Please indicate the quality of language in the manuscript: Acceptable

**Declaration of Competing Interests**

Please complete a declaration of competing interests, considering the following questions:

- Have you in the past five years received reimbursements, fees, funding, or salary from an organisation that may in any way gain or lose financially from the publication of this manuscript, either now or in the future?
- Do you hold any stocks or shares in an organisation that may in any way gain or lose financially from the publication of this manuscript, either now or in the future?
- Do you hold or are you currently applying for any patents relating to the content of the manuscript?
- Have you received reimbursements, fees, funding, or salary from an organization that holds or has applied for patents relating to the content of the manuscript?
- Do you have any other financial competing interests?
- Do you have any non-financial competing interests in relation to this paper?

If you can answer no to all of the above, write 'I declare that I have no competing interests' below. If your reply is yes to any, please give details below.

I declare that I have no competing interests.

I agree to the open peer review policy of the journal. I understand that my name will be included on my report to the authors and, if the manuscript is accepted for publication, my named report including any attachments I upload will be posted on the website along with the authors' responses. I agree for my report to be made available under an Open Access Creative Commons CC-BY license (http://creativecommons.org/licenses/by/4.0/). I understand that any comments which I do not wish to be included in my named report can be included as confidential comments to the editors, which will not be published.

I agree to the open peer review policy of the journal

To further support our reviewers, we have joined with Publons, where you can gain additional credit to further highlight your hard work (see: https://publons.com/journal/530/gigascience). On publication of this paper, your review will be automatically added to Publons, you can then choose whether or not to claim your Publons credit. I understand this statement.

Yes
