# Supplementary material for: GeneSeqToFamily: a Galaxy workflow to find gene families based on the Ensembl Compara GeneTrees pipeline
Source: Gigascience. 2018 Feb 7;7(3):giy005. doi: 10.1093/gigascience/giy005 (PMC5863215; doi:10.1093/gigascience/giy005)
Supplement: Reviewer_2_Report_(Original_Submission) -- Yvan Le Bras — 28 Apr 2017 Reviewed [file giy005_reviewer_2_report_(original_submission).docx]

**Reviewer Report**

**Title:** GeneSeqToFamily: a Galaxy workflow to find gene families based on the Ensembl Compara GeneTrees

**Version:** Original Submission	**Date:** 28 Apr 2017

**Reviewer name:** Yvan Le Bras

**Reviewer Comments to Author:**

The article presents GeneSeqToFamily, the Ensembl Compara GeneTrees pipeline as a Galaxy workflow. Authors introduce the importance of studying homologous genes to understand evolution of gene families. They present the Ensembl Compara GeneTrees pipeline and the resulting conversion into a Galaxy workflow named GeneSeqToFamily. This workflow is thus the first "ready-to-use" solution providing information about structural changes within genes using Galaxy. The article is well written and I would like to see it published, I have provided very few notes, comments, and suggestions below.
I don't succeed to find typo or other little errors in the manuscript. Moreover, I don't have any major recommendation. Only few minors remarks, maybe ideas who can be of interest… or not ;)

Article

I particularly like the manner authors have presented workflow tools in the table 1. This is a good manner to summarized components, details and origins of each tool. Moreover, this translate the wish of transparency by authors and the fact they don't want to reinvent the wheel using, if relevant, existing tools.
1) It appears to me that selecting the longest CDS per gene is a good idea and I'm wondering if this step cannot be made through the use of "classical" existing Galaxy tools as "Text manipulation" ones.
Concerning clustering steps, I want to propose some ideas, but not sure these are appropriate….
2) Not sure this is relevant but concerning clustering of similar sequences, I'm wondering if the use of algo like Swarm (https://github.com/torognes/swarm) (dedicated to metagenomics amplicon-based ngs data clustering) can be of interest…
3) Concerning the overall clustering approach and related filtered steps, maybe it can be applied a) a combination of parallel clustering methods (K-means/PAM/CAH/…) to compare "raw" clustering results and thus eliminate / reduce potential noise (ie sequences moving from one cluster to another when we change the clustering method, translating that this sequence is hard to assign) b) a clustering affecting bootstrap confidence score to nodes thus giving ideas about relevance of each cluster.
Concerning TreeBeST input datatypes,
4) you propose the use of the Newick format. Maybe, as for the Philoviz visualization plugin, it would be interesting to add Nexus and phyloxml datatypes?
I especially enjoyed the sentence about "unique genes" discovery and the fact that results can be due to real absence of homologs, incomplete annotation or parameter stringence, choice. I think this kind of "warning" is important for the users!
5) Authors mention the use of "stringent" vs "relaxed parameters" for BlastP pre-clustering step. This is something important in my opinion, and well implemented and presented. I think that the relevance of resulting gathering between the 2 approaches (ie a lot of small families vs a few of big families) can be evaluate and maybe corrected if something like 3) is applied. Once again, please, don't take this into account if 3) is not relevant!
The "Example use case" section is a good idea!
6) Maybe authors can give more details more aspects related to particular issues related to a) alternative transcripts as it appears to me that ""just"" changing the max_target_seqs value from 3 to 4 seems to be quite "light" and b) working on non-model organisms.
I particularly appreciate mention of the wish of a "community driven approach" to know successful combination of workflow parameters!
Future directions seem to be perfectly appropriate in my opinion.

Tests of the GeneSeqToFamily workflow

Unfortunately, I don't have taking time to implement the GeneSeqToFamily tools and workflow to a dedicated Galaxy instance. I would have appreciated to have a link giving access to a testing GeneSeqToFamily Galaxy instance…
7) Maybe creating and pointing a dedicated Docker based Galaxy flavor can be of interest, thus giving an easy way to deploy a GeneSeqToFamily local Galaxy instance.
8) Moreover, regarding the https://github.com/TGAC/earlham-galaxytools/tree/master/workflows/GeneSeqToFamily github link, the readme file "only" explain GeneSeqToFamily workflow without instructions on how to deploy it.
9) Regarding tools on the toolshed, it would be nice to have a repository called "suite_GeneSeqToFamily" or something like that to easily install all GeneSeqToFamily related Galaxy tools without installing it one by one like for the "suite_stacks" tools for example.

**Level of Interest**

Please indicate how interesting you found the manuscript: An article of importance in its field

**Quality of Written English**

Please indicate the quality of language in the manuscript: Acceptable

**Declaration of Competing Interests**

Please complete a declaration of competing interests, considering the following questions:

- Have you in the past five years received reimbursements, fees, funding, or salary from an organisation that may in any way gain or lose financially from the publication of this manuscript, either now or in the future?
- Do you hold any stocks or shares in an organisation that may in any way gain or lose financially from the publication of this manuscript, either now or in the future?
- Do you hold or are you currently applying for any patents relating to the content of the manuscript?
- Have you received reimbursements, fees, funding, or salary from an organization that holds or has applied for patents relating to the content of the manuscript?
- Do you have any other financial competing interests?
- Do you have any non-financial competing interests in relation to this paper?

If you can answer no to all of the above, write 'I declare that I have no competing interests' below. If your reply is yes to any, please give details below.

I declare that I have no competing interests

I agree to the open peer review policy of the journal. I understand that my name will be included on my report to the authors and, if the manuscript is accepted for publication, my named report including any attachments I upload will be posted on the website along with the authors' responses. I agree for my report to be made available under an Open Access Creative Commons CC-BY license (http://creativecommons.org/licenses/by/4.0/). I understand that any comments which I do not wish to be included in my named report can be included as confidential comments to the editors, which will not be published.

I agree to the open peer review policy of the journal

To further support our reviewers, we have joined with Publons, where you can gain additional credit to further highlight your hard work (see: https://publons.com/journal/530/gigascience). On publication of this paper, your review will be automatically added to Publons, you can then choose whether or not to claim your Publons credit. I understand this statement.

Yes
